# Distinct domains of the AVRPM3^A2/F2^ avirulence protein from wheat powdery mildew are involved in immune receptor recognition and putative effector function

**DOI:** 10.1111/nph.15026

**Published:** 2018-02-17

**Authors:** Kaitlin Elyse McNally, Fabrizio Menardo, Linda Lüthi, Coraline Rosalie Praz, Marion Claudia Müller, Lukas Kunz, Roi Ben‐David, Kottakota Chandrasekhar, Amos Dinoor, Christina Cowger, Emily Meyers, Mingfeng Xue, Fangsong Zeng, Shuangjun Gong, Dazhao Yu, Salim Bourras, Beat Keller

**Affiliations:** ^1^ Department of Plant and Microbial Biology University of Zürich Zollikerstrasse 107 8008 Zürich Switzerland; ^2^ Institute of Plant Science ARO‐Volcani Center 50250 Bet Dagan Israel; ^3^ Department of Plant Pathology and Microbiology The Robert H. Smith Faculty of Agriculture, Food and Environment The Hebrew University of Jerusalem Rehovot 76100 Israel; ^4^ United States Department of Agriculture‐Agricultural Research Service (USDA‐ARS) North Carolina State University Raleigh NC 27695 USA; ^5^ Department of Plant Pathology North Carolina State University Raleigh NC 27695 USA; ^6^ Institute of Plant Protection and Soil Science Hubei Academy of Agricultural Sciences 430064 Wuhan China; ^7^ Ministry of Agriculture Key Laboratory of Integrated Pest Management in Crops in Central China 430064 Wuhan China; ^8^ College of Life Science Wuhan University 430072 Wuhan China

**Keywords:** avirulence gene, *Blumeria graminis*, gene synthesis, natural diversity, *Nicotiana benthamiana*, *Pm3*, site‐directed mutagenesis, wheat

## Abstract

- Recognition of the AVRPM3^A2/F2^ avirulence protein from powdery mildew by the wheat PM3A/F immune receptor induces a hypersensitive response after co‐expression in *Nicotiana benthamiana*. The molecular determinants of this interaction and how they shape natural *AvrPm3* ^*a2/f2*^ allelic diversity are unknown.
- We sequenced the *AvrPm3* ^*a2/f2*^ gene in a worldwide collection of 272 mildew isolates. Using the natural polymorphisms of *AvrPm3* ^*a2/f2*^ as well as sequence information from related gene family members, we tested 85 single‐residue‐altered AVRPM3^A2/F2^ variants with PM3A, PM3F and PM3F^L^ ^456P/Y458H^ (modified for improved signaling) in *Nicotiana benthamiana* for effects on recognition.
- An intact *AvrPm3* ^*a2/f2*^ gene was found in all analyzed isolates and the protein variant recognized by PM3A/F occurred globally at high frequencies. Single‐residue alterations in AVRPM3^A2/F2^ mostly disrupted, but occasionally enhanced, the recognition response by PM3A, PM3F and PM3F^L^ ^456P/Y458H^. Residues enhancing hypersensitive responses constituted a protein domain separate from both naturally occurring polymorphisms and positively selected residues of the gene family.
- These results demonstrate the utility of using gene family sequence diversity to screen residues for their role in recognition. This approach identified a putative interaction surface in AVRPM3^A2/F2^ not polymorphic in natural alleles. We conclude that molecular mechanisms besides recognition drive *AvrPm3* ^*a2/f2*^ diversification.

## Introduction

Innate immune responses are crucial for early and efficient detection of infectious pathogens. Plant defense responses against pathogen‐caused diseases rely heavily on immune receptors that perceive infection and elicit a localized cell death response to prevent pathogen growth and proliferation. The immunity conferred by such receptors represents an important source of resistance in agriculture. In particular, intracellular nucleotide‐binding, leucine‐rich repeat receptors (NLRs) mediate resistance by detecting secreted pathogen effectors and activating effector‐triggered immune responses (effector‐triggered immunity, ETI) (Qi & Innes, 2013; Zipfel, 2014). ETI is an induced localized cell death, or hypersensitive response (HR), that is especially effective against biotrophic fungal and oomycete pathogens, which need living host tissue to survive.

To complete their life cycle, biotrophic pathogens must establish close associations with the host to acquire nutrients and avoid triggering the immune system (Koeck *et al*., 2011). To this end, filamentous fungal and oomycete pathogens secrete small effector proteins to inhibit host defense responses or hijack the cellular metabolism (Lo Presti *et al*., 2015). Fungal and oomycete effectors typically have no predicted homology to known proteins and can be structurally related, but often are not sequence related (Win *et al*., 2012; Maqbool *et al*., 2015; Lu *et al*., 2016; Praz *et al*., 2017). Pathogen fitness can depend heavily on effector function, where the loss or inactivation of important effectors reduces virulence (Huang *et al*., 2006; Bos *et al*., 2010). The recognition of specific effectors by host immune receptors also compromises fitness. Accordingly, effectors that are recognized by resistance (*R*) genes become avirulence factors (*Avr*s) and experience strong selection pressure to evade host recognition.

Interactions between host *R* genes and pathogen *Avr*s are highly specific. This specificity of recognition is considered the main driver of *Avr* allelic diversification. Examination of *Avr* natural diversity and the effects of individual polymorphisms on recognition has been highly informative for the study of *R* gene specificity. For example, resistance against *Leptosphaeria maculans* is mediated by the dual recognition of the *AvrLm4‐7* effector by both *Rlm4* and *Rlm7* resistance genes (Parlange *et al*., 2009). Isolates virulent on *Rlm4* contain *AvrLm4‐7* alleles with a single common residue alteration which has been functionally validated as specifically disrupting *Rlm4* recognition (Parlange *et al*., 2009; Blondeau *et al*., 2015). Following gain‐of‐virulence mutations in pathogens, reciprocal evolution of host *R* genes may re‐establish recognition and resistance. This has also been found in the step‐wise evolution of specificity between *Avr‐Pik* alleles from *Magnaporthe oryzae* and *Pik* alleles from rice (*Oryza sativa*) (Kanzaki *et al*., 2012). In contrast with gain‐of‐virulence on *Rlm4*,* L. maculans* isolates virulent on *Rlm7* primarily contain either deletions or several polymorphisms in *AvrLm4‐7* that lead to inactivation of the protein (Daverdin *et al*., 2012; Blondeau *et al*., 2015). This demonstrates that, in addition to diversification of the AVR protein sequence, successful evasion of recognition can also be mediated by *Avr* deletion or gene inactivation, despite potential fitness consequences (Huang *et al*., 2006, 2010).

Studies of recognition specificity have focused extensively on information from natural polymorphisms in both *Avr* and *R* genes. Variants of the avirulence factor ATR1 from *Hyaloperonospora arabidopsis* are recognized by different alleles of the RPP1 immune receptor from *Arabidopsis* (Krasileva *et al*., 2010). An informed selection of mutations from among 69 polymorphisms in ATR1 variants from eight isolates revealed that distributed recognition surfaces on ATR1 determine the specificity of different RPP1 alleles (Krasileva *et al*., 2010; Chou *et al*., 2011). Similarly, in the study of the recognition specificity of flax (*Linum usitatissimum*) *L5* and *L6* alleles for *AvrL567* variants of the flax rust pathogen (*Melampsora lini*), tests combining several mutations from among 35 naturally polymorphic sites at the *AvrL567* locus in six rust strains revealed their additive effect on recognition, suggesting that multiple amino acid contact points also determine specificity in this interaction (Ravensdale *et al*., 2012). However, only four AVR‐Pik variants were identified in a worldwide screen of 39 *M. oryzae* isolates (Kanzaki *et al*., 2012), demonstrating that, even in larger worldwide diversity screens, limited sequence diversity is often observed in fungal *Avr* genes. Therefore, *in vitro*‐created sequence diversity would be useful for determining the basis of recognition specificity for fungal *Avr*–*R* interactions.

Active *R* genes are known to coevolve with their cognate pathogen *Avr*s under strong diversifying selection or, more rarely, by balancing selection. They often form larger gene families and complex clusters of gene paralogs, but rarely form true allelic series conferring race specificity (Ellis *et al*., 1999; Rose *et al*., 2004; Seeholzer *et al*., 2010; Kanzaki *et al*., 2012). Early descriptions of the cognate *Avr*s of multiallelic race‐specific *R* genes indicated that allelic variants recognize naturally occurring AVR variants in the pathogen (Dodds *et al*., 2006; Krasileva *et al*., 2010; Kanzaki *et al*., 2012). However, recent advances in the study of multiallelic *R* genes conferring resistance to the cereal powdery mildews (*Blumeria graminis* ff. spp.), specifically the *Pm3* gene in wheat (*Triticum aestivum*) and the *Mla* gene in barley (*Hordeum vulgare*), suggest that distinct resistance alleles recognize highly sequence diverse effectors (Bourras *et al*., 2015; Lu *et al*., 2016).

Powdery mildew of wheat is caused by the obligate biotrophic fungal pathogen, *Blumeria graminis* f. sp. *tritici* (*B.g. tritici*). *B.g. tritici* followed the spread of wheat cultivation to all major wheat‐growing regions worldwide. The multiallelic *Pm3* gene from wheat encodes a coiled‐coiled (CC)‐NLR protein that confers race‐specific resistance against *B.g. tritici*. The 17 functionally distinct alleles of *Pm3* in the hexaploid bread wheat gene pool share particularly high sequence identity (>97%) (Yahiaoui *et al*., 2004; Srichumpa *et al*., 2005; Bhullar *et al*., 2010). The *Pm3a* and *Pm3f* alleles share overlapping recognition spectra towards powdery mildew races, where the spectrum of races recognized by *Pm3a* includes those recognized by *Pm3f* (Brunner *et al*., 2010). This overlap is the result of their shared specificity for the *AvrPm3* ^*a2/f2*^ gene encoded by *B.g. tritici*. The *AvrPm3* ^*a2/f2*^ gene belongs to a family of 24 sequence‐divergent, but structurally related, secreted effectors (Bourras *et al*., 2015). In transient expression assays in *Nicotiana benthamiana*, it was demonstrated that AVRPM3^A2/F2^ is recognized specifically by the *Pm3a* and *Pm3f* alleles. The HR elicited by PM3A recognition is much stronger than that elicited by the PM3F allele; however, it was demonstrated that two substitutions (L456P, Y458H) in the ARC2 subdomain of the nucleotide‐binding site domain (NBS) of PM3F are sufficient to enhance this response to levels comparable with those of PM3A (Stirnweis *et al*., 2014). In contrast with gene‐for‐gene interactions (Flor, 1971), segregating phenotypes among the progeny of a genetic cross of isolates hinted at the involvement of a second pathogen‐encoded factor, the suppressor‐of‐recognition *SvrPm3* ^*a1/f1*^, which was cloned and functionally validated. Expression analyses of both fungal effector genes suggested that the *SvrPm3* ^*a1/f1*^ suppressor acts quantitatively to suppress *AvrPm3* ^*a2/f2*^ recognition mediated by PM3A/F (Bourras *et al*., 2015). Altogether, these findings led to the development of the *Avr‐R‐Svr* model of interaction in the wheat–powdery mildew pathosystem, where *R* specificity for *Avr* recognition is modified by the action of an *Svr* (Bourras *et al*., 2016).

In this study, we examined the basis of *Pm3a/f* recognition specificity using the natural sequence diversity of *AvrPm3* ^*a2/f2*^ from a worldwide collection of *B.g. tritici* and *B.g. triticale*. We found that *AvrPm3* ^*a2/f2*^ is present in all isolates and shows limited natural diversity worldwide. We provide further evidence of the role of the suppressor *SvrPm3* ^*a2/f2*^ in increasing the virulence of isolates from Europe that express the active *AvrPm3* ^*a2/f2*^. Using the sequence diversity from the structurally related *AvrPm3* ^*a2/f2*^ effector family, we identified a region of AVRPM3^A2/F2^ in which mutations strongly influence recognition and specificity. This putative interaction domain does not overlap with residues under positive selection in the effector family or residues polymorphic in the natural isolates. In light of our results, we conclude that using sequence diversity from a related gene family is informative for studies of recognition specificity, and propose that, for the *AvrPm3* ^*a2/f2*^–*Pm3a/f* interaction, selection pressure from recognition is not the primary source of *AvrPm3* ^*a2/f2*^ allelic diversity.

## Materials and Methods

### Fungal collection, propagation and virulence tests

*Blumeria graminis* isolates were maintained on detached leaves of the appropriate susceptible cereal cultivar (‘Kanzler’ (*Triticum aestivum*), ‘Inbar’ (*Triticum durum*) or ‘Matador’ (*Triticosecale*)) on benzimidazole agar, as described by Parlange *et al*. (2011). The worldwide collection contained 272 isolates: 55 isolates collected in 13 states in the USA (Cowger *et al*., 2018), 101 collected in 12 provinces in China (Zeng *et al*., 2014), 61 collected in four eco‐geographic regions of Israel (Ben‐David *et al*., 2016), 51 collected primarily in Switzerland as well as in five other European countries (our collection), two in Japan and two in Australia. All isolates from Europe, Japan and Australia, and a subset of isolates from the USA, China and Israel, were maintained in‐house. Spore samples for DNA extraction and sequencing by PCR were obtained primarily for US and Israeli isolates, whereas complete genomes for a subset of Chinese isolates were used for *in silico* extraction (Praz *et al*., 2017). European, Japanese and Australian isolates were collected in the field and single spore isolated twice, as described by Brown & Wolfe (1990). Virulence tests on *Pm3a* (cv. Asosan/8*Chancellor) and *Pm3f* (cv. Michigan Amber/8*Chancellor) of isolates in‐house were performed as described by Brunner *et al*. (2010), and scored as described in Bourras *et al*. (2015). External Israeli, US and Chinese isolates were collected, maintained and phenotyped as described in Ben‐David *et al*. (2016) (Israeli and US) and Zeng *et al*. (2014) (Chinese).

### DNA/RNA isolation and construction of plasmid vectors

High‐molecular‐weight DNA for PCR amplification was extracted as described previously by Bourras *et al*. (2015). RNA samples were extracted from infected detached ‘Chancellor’ leaves using the Qiagen miRNeasy Mini Kit (Qiagen) according to the manufacturer. Full‐length cDNA was prepared using the Superscript III RT kit (Invitrogen) according to the manufacturer. Molecular cloning into the Gateway‐compatible entry vector was performed using the pENTR/D‐TOPO Cloning Kit (Invitrogen) according to the manufacturer. Gene synthesis including Gateway‐compatible cloning sites was performed by gen9 (https://www.gen9bio.com/), resulting in a Gateway‐compatible cloning vector (pG9m‐2) containing the synthesized gene. The synthesized gene was then cloned directly into the pIPKb004 expression vector (Himmelbach *et al*., 2007) used for transient expression in *N. benthamiana*. Site‐directed mutagenesis was performed by PCR amplification using overlapping primers containing desired mutations on the Gateway‐compatible pENTR/D‐TOPO vector containing *AvrPm3* ^*a2/f2*^. Recombination of the mutant PCR product into the binary vector pIPKb004 was performed as described previously by Stirnweis *et al*. (2014). Primers used for gene amplification and site‐directed mutagenesis are listed in Supporting Information Table S1. Constructs are listed in Table S2. All *AvrPm3* ^*a2/f2*^ and *SvrPm3* ^*a1/f1*^ genomic sequences used for this study are available at the GenBank database under the accession numbers MG739404–MG739429.

### Genetic analyses and tests for selection

The Templeton, Crandall and Sing (TCS) network was visualized using the popart software (Leigh & Bryant, 2015). Maximum likelihood computations and estimation of the average nonsynonymous/synonymous (*d*_N_ */d*_S_) rate ratio for *AvrPm3* ^*a2/f2*^ haplotypes were conducted using the hyphy software package (Pond *et al*., 2005) in the mega7 program (Kumar *et al*., 2016). All multiple alignments were performed with muscle 3.8.31 (Edgar, 2004). The protein alignments were back‐translated to nucleotide alignments using translatorx v1.1 (Abascal *et al*., 2010). To test for positive selection, we estimated the likelihood of the maximum likelihood tree under the M8a and M8 models (Yang & Nielsen, 2000) with paml 4.8 (Yang, 2007) and using the Bayes empirical Bayes method (Yang *et al*., 2005).

### Quantitative real‐time PCR experiments

Quantitative real‐time PCR experiments were performed as described in Praz *et al*. (2017) with the modification of a 20‐s extension time. Three independent biological replicates were sampled at 2 d after inoculation. Glyceraldehyde 3‐phosphate dehydrogenase (*Gapdh*) was used as an internal control, as described in Bourras *et al*. (2015). Gene expression was normalized to that of *Gapdh*. Quantitative real‐time PCR primers for reference and target genes have been described previously (Bourras *et al*., 2015).

### Transient protein expression assays in *N. benthamiana*

Transient expression by agroinfiltration in *N. benthamiana* was conducted according to the protocols of Ma *et al*. (2012) and Dugdale *et al*. (2014), and with the modifications described by Bourras *et al*. (2015). To test for *AvrPm3*–*Pm3* interaction, *Agrobacterium* expressing the *AvrPm3* constructs or the *Pm* resistance allele were mixed in a 4 : 1 ratio of *Avr* : *R*. At least three replications were performed and the HR was visualized as described by Praz *et al*. (2017). HR intensity was calculated using the mean gray value estimated by ImageJ (Schneider *et al*., 2012) and normalizing against the noninfiltrated background. Normalized values were compared between HR elicited by mutant *AvrPm3* ^*a2/f2*^ constructs and wild‐type *AvrPm3* ^*a2/f2*^ constructs, and statistical significance was estimated using the Student's *t*‐test.

## Results

### A worldwide survey reveals the ubiquitous presence and limited sequence diversity of *AvrPm3* ^*a2/f2*^

To study the natural sequence diversity of AVRPM3^A2/F2^ and to characterize the variants eliciting recognition by *Pm3a/f*, we sequenced the complete *AvrPm3* ^*a2/f2*^ gene in an unprecedented worldwide collection of 272 isolates with diverse genetic backgrounds and a balanced representation of global geographic origins (Table S3). The 251 *B.g. tritici* and 22 *B.g. triticale* (powdery mildew of triticale) isolates form six geographically distinct populations: USA, 56 isolates from 13 states; China, 101 isolates from 12 provinces (Zeng *et al*., 2014); Europe, 51 isolates from six countries; Israel, 61 isolates (Ben‐David *et al*., 2016); Australia, two isolates; Japan, two isolates. All sampled isolates within this collection encoded a complete *AvrPm3* ^*a2/f2*^ gene, with no case of nonsense mutation, truncation or complete gene deletion. Although it is impossible to identify identical gene duplications by sequencing of PCR products, our whole gene sequencing data yielded no evidence of mixed gene sequences that would indicate multiple divergent *AvrPm3* ^*a2/f2*^ sequences encoded by a single isolate. In addition, *de novo* analysis of 41 available whole genome assemblies using iterative blast searches of the *AvrPm3* ^*a2/f2*^ gene sequence revealed no evidence of duplications or multiple haplotypes encoded in a single isolate. We identified 11 novel *AvrPm3* ^*a2/f2*^ haplotypes in addition to the two reported previously (Bourras *et al*., 2015). The 13 haplotypes share 95–99% identity, and 12 encode unique protein sequences (labelled A–H, J–M, Table 1). All novel protein encoding haplotypes were produced by site‐directed mutagenesis or gene synthesis and tested in transient assays in *N. benthamiana* for recognition by PM3A, PM3F and PM3F^L456P/Y458H^, as described in Bourras *et al*. (2015). We used the previously validated AVRPM3^A2/F2^‐A as the positive control for HR induction upon recognition by PM3A and PM3F^L456P/Y458H^. By visual inspection and fluorescence imaging (Praz *et al*., 2017) of the assayed *N. benthamiana* leaves at 5 d post‐infiltration, we observed that none of the new variants induced HR in the presence of PM3A or PM3F^L456P/Y458H^, suggesting that they encode inactive AVR proteins (Fig. S1). These results indicate that AVRPM3^A2/F2^‐A (Bourras *et al*., 2015) is the only active AVR variant in natural isolates.

**Table 1:** Disrupting recognition by polymorphisms in the amino acid sequences of the AVRPM3^A2/F2^ variants from wheat powdery mildew

	No.	21	24	25	26	27	31	38	52	66	69	80	86	89	91	93	95	109	119	122	123
AVRPM3^A2/F2^‐A^a^	122	A	S	G	‐	‐	N	H	E	N	R	N	G	N	K	E	F	A	Y	T	E
AVRPM3^A2/F2^‐C	28	.	.	.	‐	‐	.	.	.	.	.	.	.	.	.	.	L	.	.	.	.
AVRPM3^A2/F2^‐E	16	.	.	.	‐	‐	.	.	.	.	.	.	.	.	.	D	.	.	.	.	.
AVRPM3^A2/F2^‐F^b^	15	.	.	.	‐	‐	.	.	.	K	S	.	E	.	.	.	.	.	.	.	.
AVRPM3^A2/F2^‐D	24	.	.	.	‐	‐	.	.	.	K	.	S	.	.	T	K	.	.	.	.	.
AVRPM3^A2/F2^‐B	52	.	.	.	‐	‐	.	Q	.	.	.	.	E	.	.	.	.	.	.	.	D
AVRPM3^A2/F2^‐G	4	V	N	S	P	V	E>	Q	.	K	.	.	E	Y	.	.	.	V	H	.	.
AVRPM3^A2/F2^‐M	1	.	.	.	‐	‐	E	Q	N	.	.	.	E	.	.	.	.	.	.	.	D
AVRPM3^A2/F2^‐K	2	.	.	.	‐	‐	.	Q	.	.	.	.	E	.	.	.	.	.	.	.	.
AVRPM3^A2/F2^‐H	4	.	.	.	‐	‐	.	.	.	.	.	.	E	.	.	.	.	.	.	.	D
AVRPM3^A2/F2^‐J	3	.	.	.	‐	‐	.	.	.	.	.	.	E	.	.	.	.	.	.	R	D
AVRPM3^A2/F2^‐L^a^	1	.	.	.	‐	‐	.	.	.	.	.	.	E	.	.	.	.	V	.	.	.
Total	272																				

Polymorphisms compared with the avirulent variant are depicted using the one‐letter code for amino acids. Residue numbering includes the signal peptide. The number of isolates encoding each variant in the global collection of 272 isolates is given (No.). Residues that individually disrupt recognition by PM3A and PM3F^L456P/Y458H^ are indicated with black boxes. Untested residues are in gray boxes. Dashes indicate gaps and dots indicate identical residues.

a Bourras *et al*. (2015).

b Variant found in isolates collected on *Triticum dicoccoides* (wild emmer).

**Figure 1:**
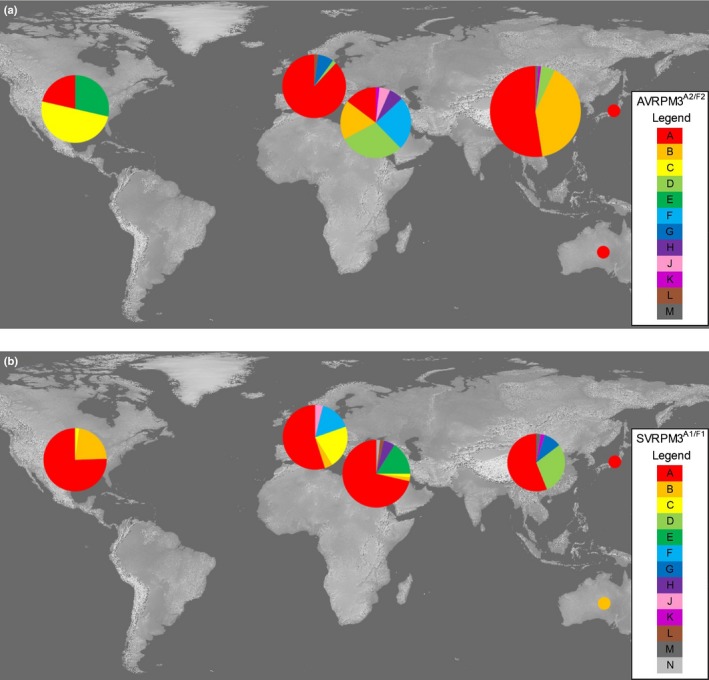
Geographic distribution of the AVRPM3^A2/F2^ and SVRPM3^A1/F1^ variants. (a) Geographic distribution of the 12 AVRPM3^A2/F2^ variants (A–H, J–M) encoded by *Blumeria graminis* f. sp. *tritici* and *triticale* isolates from the USA, Europe, Israel, China, Japan and Australia. (b) Geographic distribution of the SVRPM3^A1/F1^ variants (A–H, J–N) encoded by *Blumeria graminis* f. sp. *tritici* and *triticale* isolates from the USA, Europe, Israel, China, Japan and Australia. The areas of the circles are proportional to the number of isolates from that region in the tested collection.

Genotyping the mildew collection revealed that the *AvrPm3* ^*a2/f2*^ *‐A* haplotype is the most common worldwide (122 isolates, 45%), and is present in all six geographic populations at varying frequencies (15–88%, excluding Australia and Japan; Fig. 1a). We obtained phenotypic data on both *Pm3a* and *Pm3f* wheat for 166 isolates in the collection (Table 2). Comparing *AvrPm3* ^*a2/f2*^ genotypes and isolate phenotypes on *Pm3a* and *Pm3f*, we observed that, of the isolates encoding any of the inactive AVRPM3^A2/F2^ variants B–H, J–M, 72% (65/90) were virulent on *Pm3a*, whereas 78% (70/90) were virulent on *Pm3f* (Table 2). However, all isolates encoding variant ‘F’ (also not recognized in our transient assay) were consistently avirulent on *Pm3a* and *Pm3f*, with one exception. We also tested the hypothesis that variant ‘F’ is recognized by other *Pm3* alleles using the same assays in *N. benthamiana*. We found no evidence of interaction with *Pm3b*,* Pm3c*,* Pm3d* and *Pm3e* alleles, or with the mildew resistance gene *Pm8* (Fig. S2). Evidence of a third factor (*AvrPm3* ^*a3*^) recognized by *Pm3a* was found previously in a genetic cross of two mildew isolates segregating on *Pm3a* and *Pm3f* (Bourras *et al*., 2015). There is also genetic evidence from one UV‐mutagenized mildew strain (EXP3) that a fourth factor regulates *AvrPm3* ^*a2/f2*^ gene expression (Bourras *et al*., 2015; Parlange *et al*., 2015). Therefore, it is likely that additional *Avr* factors or *Avr* modifiers polymorphic in global populations could explain the avirulence of isolates harboring AVRPM3^A2/F2^‐F on *Pm3a/f*. Another possibility is that *AvrPm3* ^*a2/f2*^ is recognized by another NLR in wheat. Although evidence from our genetic mapping populations indicates that *AvrPm3* ^*a2/f2*^ is not recognized by *Pm1a*,* Pm2*,* Pm3b‐e*,* Pm3g*,* Pm4a*,* Pm4b*,* Pm5a*,* Pm8* and *Pm17,* we cannot exclude possible recognition by NLRs other than those we have tested (Parlange *et al*., 2015; Bourras *et al*., 2015; Praz *et al*., 2017; Notes S1; Tables [Link], [Link], [Link]). In isolates encoding the recognized *AvrPm3* ^*a2/f2*^ *‐A* haplotype, we observed that 71% (54/76) were avirulent on *Pm3a* wheat, and 17 of these (22%) were also avirulent on *Pm3f* wheat (Table 2). Together, these results suggest that additional pathogen‐encoded factors besides *AvrPm3* ^*a2/f2*^ are contributing to recognition by *Pm3a/f* in natural mildew populations.

**Table 2:**
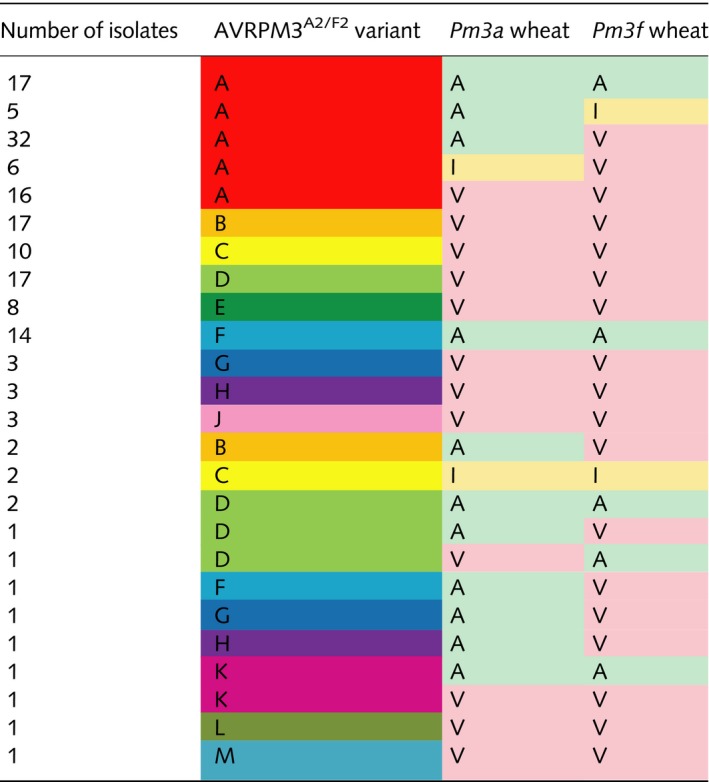
Summary of AVRPM3^A2/F2^ variants and phenotypes on *Pm3a* and *Pm3f* wheat observed in natural isolates

AVRPM3^A2/F2^ variants (A–H, J–M) found in natural *Blumeria graminis* f. sp. *tritici* and *triticale* isolates are listed, together with the observed phenotypes on *Pm3a* and *Pm3f* wheat. Variant A (red) is recognized by PM3A and PM3F^L456P/Y458H^ in functional assays. Phenotypes (avirulent, A (green); intermediate, I (yellow); virulent, V (pink)) are colored for ease of reading.

To summarize, our results reveal limited natural diversity of *AvrPm3* ^*a2/f2*^, and the prevalence of the AVRPM3^A2/F2^‐A protein variant, suggesting an important role of *avr/AvrPm3* ^*a2/f2*^ and, in particular, the active AVRPM3^A2/F2^‐A allele in pathogen fitness. In addition, our phenotypic data suggest that other factors are involved in the interaction. For example, an as yet uncharacterized *AvrPm3* ^*a3*^ avirulence gene might be rendering isolates without the active AVRPM3^A2/F2^‐A variant avirulent on *Pm3a*, whereas the suppressor of recognition, *SvrPm3* ^*a1/f1*^, is probably increasing the virulence in isolates encoding the active AVRPM3^A2/F2^‐A variant.

### Virulence in isolates encoding the recognized AVRPM3^A2/F2^‐A is associated with the SVRPM3^A1/F1^ suppressor

Our phenotypic data indicate that, of the 54 of 76 isolates encoding the AVRPM3^A2/F2^‐A variant that are virulent on *Pm3f*, 16 are also virulent on *Pm3a* despite the presence of the recognized *AvrPm3* ^*a2/f2*^ allele (Table 2). Previously, a suppressor of recognition, *SvrPm3* ^*a1lf1*^, was functionally validated as quantitatively suppressing the recognition of *AvrPm3* ^*a2/f2*^ by *Pm3a/f* in a genetic cross of two European mildew isolates (Bourras *et al*., 2015). Polymorphic presence of the suppressor might cause the high variability of virulence in isolates encoding the active AVRPM3^A2/F2^‐A variant on *Pm3a* and *Pm3f* wheat. To determine whether the presence of the active suppressor is associated with virulence, we obtained sequences of the *SvrPm3* ^*a1/f1*^ gene from 201 isolates from the worldwide collection (for which genomic DNA or genome sequences were available), and compared the *Svr* genotypes and phenotypes of the subset of 76 isolates encoding the active *AvrPm3* ^*a2/f2*^. As with *AvrPm3* ^*a2/f2*^, we found a complete *SvrPm3* ^*a1/f1*^ gene in all isolates, suggesting that it also contributes to pathogen fitness. We identified seven new haplotypes in addition to the six haplotypes described previously (Parlange *et al*., 2015), for a total of 13 unique, but highly similar, protein variants (98–99% sequence identity; labeled A–H, J–N, Table 3). The previously reported active suppressor variant, SVRPM3^A1/F1^‐A, is also the most predominant (130 isolates) globally (Fig. 1b). For 155 isolates, we obtained both *AvrPm3* ^*a2/f2*^ and *SvrPm3* ^*a1/f1*^ sequences, as well as phenotypic data on *Pm3a* and *Pm3f* wheat (Table 4). Of these isolates, 96 encode the active suppressor and, among these, 91 are virulent on *Pm3f* and 68 are virulent on *Pm3a*. Strikingly, with only one exception, all isolates encoding the recognized AVRPM3^A2/F2^‐A variant and the active suppressor variant SVRPM3^A1/F1^‐A (42 isolates) show virulence or intermediate virulence on *Pm3f*. By contrast, all isolates encoding AVRPM3^A2/F2^‐A and suppressor variants other than SVRPM3^A1/F1^‐A (32 isolates) are avirulent on *Pm3a*, with two exceptions (Table 4), suggesting that most SVR variants other than ‘A’ do not function as active suppressors. The SVRPM3^A1/F1^‐B variant might have low suppressor activity, as we found eight isolates encoding AVRPM3^A2/F2^‐A and SVRPM3^A1/F1^‐B that were virulent on *Pm3f* wheat. These results indicate that *Pm3f* resistance can be suppressed by SVRPM3^A1/F1^‐A independently of the mildew isolate genetic background.

**Table 3:** Amino acid sequences of the SVRPM3^A1/F1^ variants in wheat powdery mildew

	No.	22	31	32	70	71	102	118	125	127
SVRPM3^A1/F1^‐A^a^	130	I	K	P	A	R	L	M	Y	T
SVRPM3^A1/F1^‐B^a^	16	.	.	.	.	H	.	.	.	.
SVRPM3^A1/F1^‐C^a^	13	.	.	.	.	H	P	.	.	.
SVRPM3^A1/F1^‐N^a^	1	.	.	H	.	H	P	.	.	.
SVRPM3^A1/F1^‐F^a^	8	.	.	.	.	H	P	.	.	K
SVRPM3^A1/F1^‐J	2	.	N	.	.	H	P	.	.	.
SVRPM3^A1/F1^‐H	3	T	.	.	.	H	P	.	.	.
SVRPM3^A1/F1^‐E^a^, ^b^	9	.	.	.	S	H	S	.	.	.
SVRPM3^A1/F1^‐D	12	.	.	.	.	H	F	.	.	.
SVRPM3^A1/F1^‐M	1	.	.	.	.	H	F	V	.	.
SVRPM3^A1/F1^‐K	1	.	.	.	.	.	F	.	.	.
SVRPM3^A1/F1^‐G	4	.	.	.	.	.	.	V	.	.
SVRPM3^A1/F1^‐L	1	.	.	.	.	.	.	.	C	.
Total	201									

Polymorphisms compared with the known active suppressor variant are depicted using the one‐letter code for amino acids. Residue numbering includes the signal peptide. Dots indicate identical residues.

a Parlange *et al*. (2015).

b Variant found in isolates collected on wild emmer (*Triticum dicoccoides*).

**Table 4:**
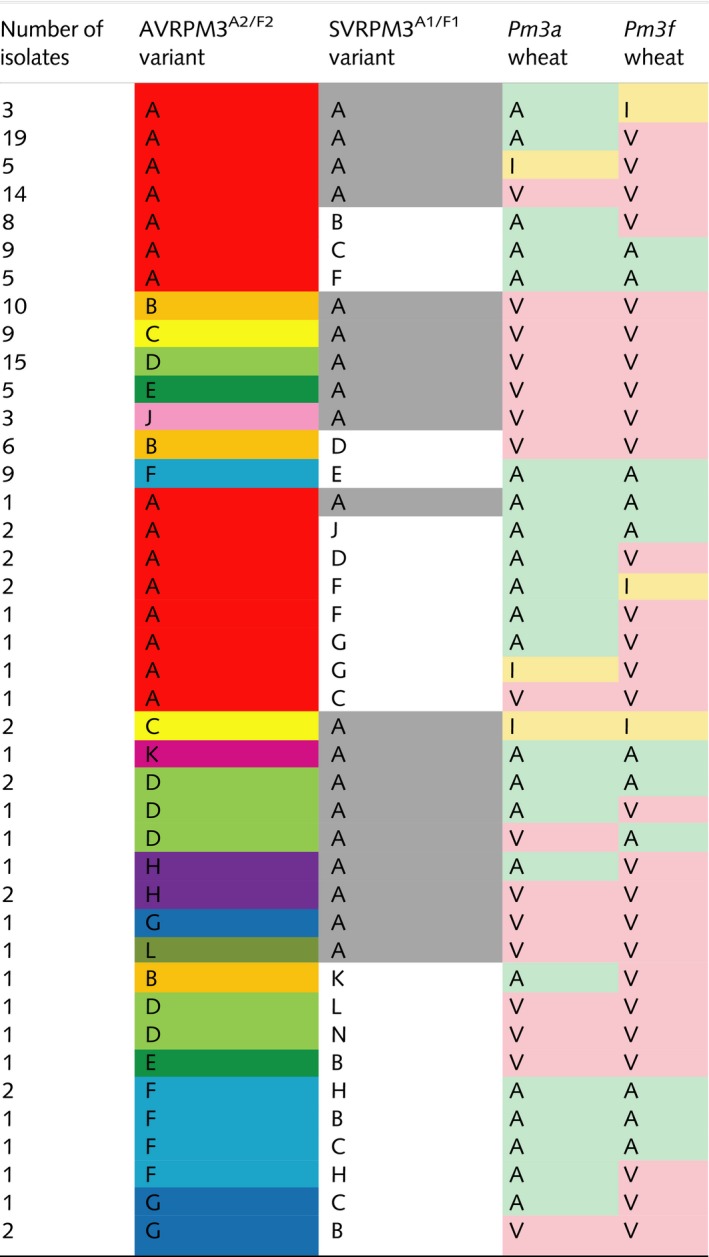
Summary of unique AVRPM3^A2/F2^ and SVRPM3^A1/F1^ variant combinations and isolate phenotypes on *Pm3a* and *Pm3f* observed in natural isolates

Unique AVR–SVR variant combinations from *Blumeria graminis* f. sp. *tritici* and *triticale* isolates and their observed phenotypes on *Pm3a* and *Pm3f* are shown. AVRPM3^A2/F2^ variants (A–H, J) and SVRPM3^A1/F1^ variants (A–F, H and J) are indicated. AVRPM3^A2/F2^‐A is the only recognized AVR variant (red), and SVRPM3^A1/F1^‐A is the known active suppressor variant (gray, Bourras *et al*., 2015). Phenotypes (avirulent, A (green); intermediate, I (yellow); virulent, V (pink)) are colored for ease of reading. Variant–phenotype combinations observed in less than three isolates are listed separately for clarity.

**Figure 2:**
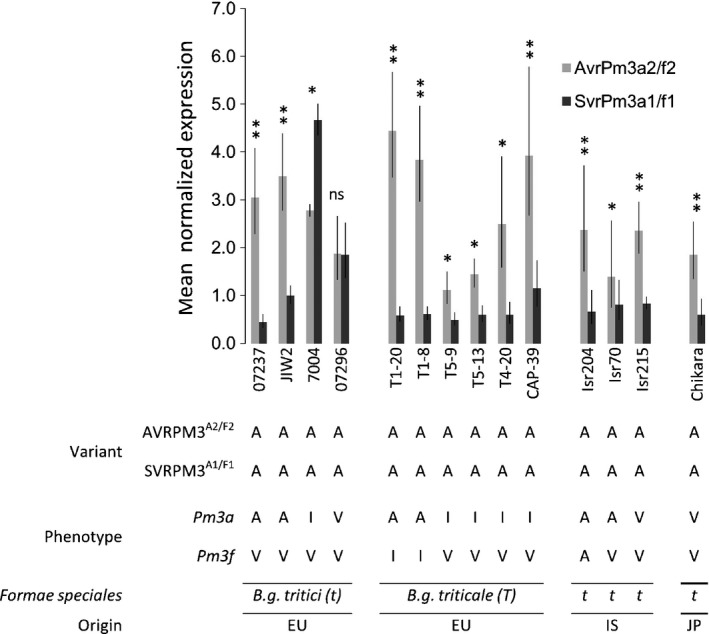
Expression analysis of isolates encoding the AVRPM3^A2/F2^‐A and SVRPM3^A1/F1^‐A variant combination which give different phenotypes on *Pm3a* and *Pm3f* wheat. The mean normalized expression of *AvrPm3* ^*a2/f2*^ (light gray histograms) and *SvrPm3* ^*a1/f1*^ (dark gray histograms) at 2 d post‐infection. Leaf segments from the susceptible recurrent parent line ‘*Chancellor*’ were infected with *Blumeria graminis* f. sp. *tritici (t)* and *B.g. triticale (T)* isolates from diverse geographic origins (EU, Europe; IS, Israel; JP, Japan) that contain the same *Avr‐Svr* genotype, but have different virulences (avirulent, A; intermediate, I; virulent, V) on *Pm3a* and *Pm3f* wheat. The results of a *t*‐test comparing *AvrPm3* ^*a2/f2*^ and *SvrPm3* ^*a1/f1*^ expression are given by * and **, indicating *P* < 0.05 and *P* < 0.01, respectively (ns, not significant). The error bars indicate ± SEM.

Most of the phenotypic variability of isolates encoding both the active AVRPM3^A2/F2^ and SVRPM3^A1/F1^ is observed in the European population in which the suppressor was originally described. It is also the population in which the combination of both active AVRPM3^A2/F2^ and SVRPM3^A1/F1^ is most frequent (24/50 isolates, 48%, Fig. S3). Within the European population, we identified 12 isolates that combine both active variants, but are avirulent on *Pm3a* wheat (Table S3). To test whether this phenotype can be explained by low expression of the suppressor gene, we compared the gene expression levels of *AvrPm3* ^*a2/f2*^ *‐A* and *SvrPm3* ^*a1/f1*^ *‐A* in two *Pm3a* virulent European *B.g. tritici* isolates (7004 and 07296) and two *Pm3a* avirulent European *B.g. tritici* isolates (07237 and JIW2) at 2 d post‐infection (Fig. 2). The isolates virulent on *Pm3a* wheat (7004 and 07296) showed similar (07296) or higher (7004) expression levels of *SvrPm3* ^*a1/f1*^ *‐A* relative to *AvrPm3* ^*a2/f2*^ *‐A*, consistent with the hypothesis that virulence in these isolates is mediated by relatively higher *Svr* expression. In the *Pm3a* avirulent isolates, we found that *SvrPm3* ^*a1/f1*^ *‐A* was expressed at significantly lower levels than *AvrPm3* ^*a2/f2*^ *‐A*, and also relative to the expression of the suppressor in the *Pm3a* virulent isolates, 7004 and 07296 (Fig. 2; Table S7). These results substantiate our hypothesis that avirulence on *Pm3a*, in the subset of 12 European isolates combining active AVRPM3^A2/F2^ and SVRPM3^A1/F1^ variants, can be explained by lower expression levels of *SvrPm3* ^*a1/f1*^ *‐A*. In addition, we compared gene expression levels of *AvrPm3* ^*a2/f2*^ *‐A* and *SvrPm3* ^*a1/f1*^ *‐A* in six European *B.g. triticale* isolates, three Israeli *B.g. tritici* isolates and one Japanese *B.g. tritici* isolate with various phenotypes on *Pm3a* and *Pm3f* (Fig. 2). We observed some association between *SvrPm3* ^*a1/f1*^ *‐A* expression relative to *AvrPm3* ^*a2/f2*^ *‐A* expression and virulence in the *B.g. triticale* isolates, but no association in isolates from Israel and Japan. These data suggest that the quantitative action of a suppressor might be most observable in the European population, and that other factors known to be involved in the interaction (e.g. *AvrPm3* ^*a3*^) might prevent observable associations between phenotype and *SvrPm3* ^*a1/f1*^ *‐A* expression in other populations. In an analysis of the expression of *AvrPm3* ^*a2/f2*^ *‐A* with other *SvrPm3* ^*a1/f1*^ haplotypes (*B*,* C*,* D*,* F*,* G* and *J*), we did not observe any similar association between expression and virulence, although expression of these variants was overall lower than *SvrPm3* ^*a1/f1*^ *‐A,* and appeared to be more consistent within isolates and less polymorphic between isolates encoding the same haplotype (Fig. S4). Taken together, our results demonstrate that recognition of *AvrPm3* ^*a2/f2*^ *‐A* encoding isolates by *Pm3a/f* wheat is strongly associated with the presence and expression level of *SvrPm3* ^*a1/f1*^ *‐A* in European isolates, thus providing additional evidence for the *Avr‐R‐Svr* genetic model at the population level.

### Single‐residue changes in AVRPM3^A2/F2^ are sufficient to abolish, enhance or alter recognition by *PM3A*,* PM3F* and *PM3F* ^*L456P/Y458H*^

**Figure 3:**
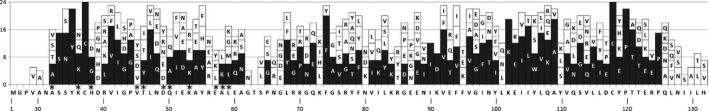
Conserved residues and motifs within the AVRPM3^A2/F2^ family from wheat powdery mildew. The protein sequences of 24 family members without the signal peptide were aligned and compared with the AVRPM3^A2/F2^ sequence. Residues present in at least three family members (white histograms) and the most frequent residues conserved in more than six of 24 family members (black histograms) are shown. Asterisks indicate residues under diversifying selection (probability (ω > 1) of > 0.95).

In the study of the recognition specificity of *L5* and *L6* for *AvrL567*, Ravensdale *et al*. (2012) chose a subset of four residues among 35 polymorphic sites associated with differences in specificity among *AvrLm567* alleles, and tested their individual contribution towards specificity. More recently, Maqbool *et al*. (2015) used protein structural information to design four mutations in AVR‐PikD which they predicted would disturb binding to the PikP cognate NLR. As neither excessive natural polymorphisms nor protein structural information was available for AVRPM3^A2/F2^, our strategy was to combine information from the 17 natural polymorphisms with an analysis of sequence diversity from the previously described *AvrPm3* ^*a2/f2*^ effector family (Bourras *et al*., 2015). AVRPM3^A2/F2^ belongs to a family of 24 small effectors (90–111 residues in the mature peptide) sharing very little sequence similarity, but an overall structural homology (Bourras *et al*., 2016). We wanted to select single‐residue alterations across the entire AVRPM3^A2/F2^ protein to identify amino acid residues affecting recognition specificity by *Pm3a* and *Pm3f*. We assumed that highly conserved residues among the 24 family members were important for structure and that substitutions in AVRPM3^A2/F2^ with residues conserved in many other family members would be structurally conservative. Following this strategy for large‐scale mutagenesis that conserved protein structure, we individually altered residues in AVRPM3^A2/F2^ towards residues shared by at least six (25%) family members (Fig. 3). In addition, the three most highly conserved residues in the family, Y35, C37 and C118, were individually changed to glycine to test their role in recognition, and mutations at residues 102, 128 and 132 were included for even coverage using either the next most frequent residue or the residue encoded by the close relative, *Pu_23*.

**Figure 4:**
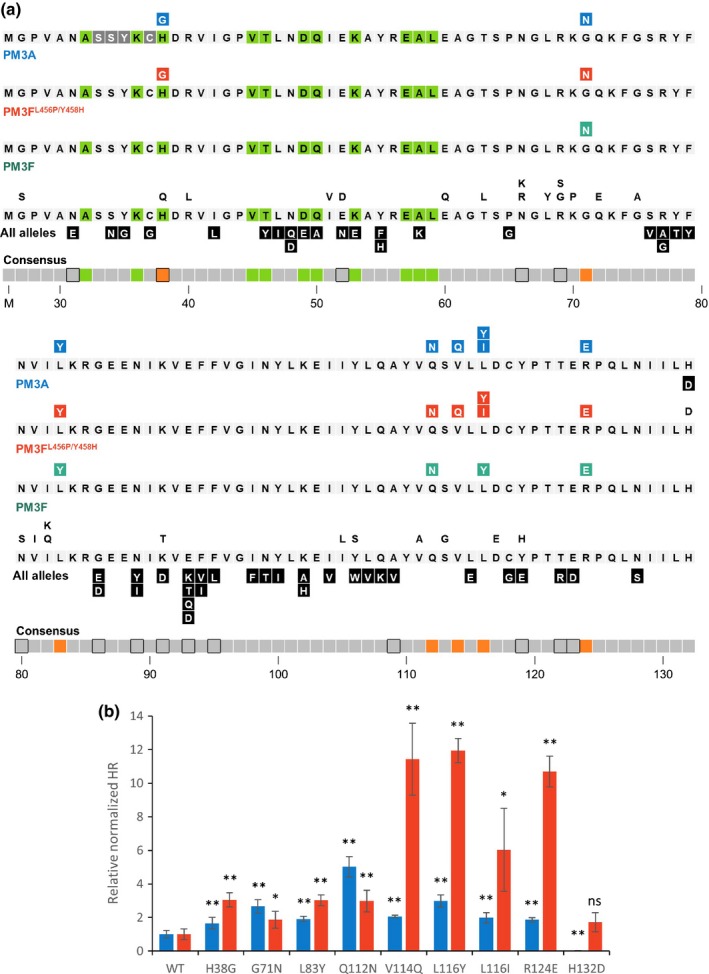
Single‐residue alterations in the wheat powdery mildew AVRPM3^A2/F2^ avirulence protein tested by co‐expression in *Nicotiana benthamiana* with the nucleotide‐binding, leucine‐rich repeat receptors (NLRs) PM3A, PM3F and PM3F^L^ ^456P/Y458H^. Single‐residue changes derived from natural polymorphisms in the variants and sequence variation in the effector family span the complete AVRPM3^A2/F2^ protein sequence without the signal peptide. These were tested by transient co‐expression in *N. benthamiana* with constructs expressing PM3A, PM3F and PM3F^L^ ^456P/Y458H^. (a) The sequence of the AVRPM3^A2/F2^ protein without signal peptide (replaced by methionine) is depicted, with the residues under diversifying selection in the family (green boxes) indicated. Results are shown on three lines separately for PM3A, PM3F^L^ ^456P/Y458H^ and PM3F. Single‐residue alterations that do not affect recognition are not colored, whereas mutations that disrupt recognition (black boxes), enhance the hypersensitive response (HR) with PM3A (blue boxes), PM3F^L^ ^456P/Y458H^ (red boxes) or gain a visible HR with PM3F (teal boxes) are indicated. A consensus diagram is given of the residues under positive selection in the family (green boxes), polymorphic residues in the variants (boxes outlined in black) and residues that result in enhanced HR when co‐expressed with PM3A, PM3F or PM3F^L^ ^456P/Y458H^ compared with the wild‐type (orange boxes). (b) The subset of constructs selected for quantification based on their enhancement or altered patterns of HR when co‐expressed with PM3A or PM3F^L^ ^456P/Y458H^. Normalized quantification of the HR of mutants co‐expressed with PM3A (blue histograms) or PM3F^L^ ^456P/Y458H^ (red histograms) is shown relative to the wild‐type (WT). Asterisks indicate significant deviation (*P < 0.05 and **P < 0.01) from the HR elicited by the wild‐type construct individually for each leaf assay using a two‐tailed Student's *t*‐test. The error bars indicate ± SEM.

Including the two single mutations described previously (Bourras *et al*., 2015), we generated 85 constructs individually mutating 66/106 residues (62.3%) with an average of 1.3 mutations per amino acid (Fig. 4a). We co‐infiltrated each of these constructs with constructs expressing PM3A, PM3F and the modified PM3F^L456P/Y458H^ in *N. benthamiana* and assessed HR at 5 d post‐infiltration. Of the 85 single‐residue‐altered constructs, 25 elicited HR to levels comparable with the wild‐type (‘neutral’ alterations), whereas a majority of alterations (50/85, 59%) elicited no HR with any construct (‘disruptive’ alterations, Fig. 4a). For the alterations that were disruptive or neutral on PM3A and PM3F^L456P/Y458H^, we did not observe HR when co‐expressed with the natural PM3F allele. At four positions, amino acid changes were either neutral or disruptive, depending on the residue chosen for replacement (positions 52, 91, 106 and 119; Fig. 4a). Interestingly, we identified a single mutation (H132D) that abolished recognition by PM3A, whilst maintaining the wild‐type‐level HR when co‐expressed with PM3F^L456P/Y458H^ (Fig. 4a,b). This is surprising as this is the final C‐terminal residue of the AVRPM3^A2/F2^ protein, and residues at the ends of proteins are not often implicated in protein–protein interactions or recognition specificity (Bos *et al*., 2010). Overall, we observed disruptive alterations across the AVRPM3^A2/F2^ protein, whereas neutral alterations were concentrated in residues 60–75 (Fig. 4a).

Surprisingly, we identified eight mutations that enhanced the HR (‘enhancing’ alterations) with either PM3A, PM3F^L456P/Y458H^, or both (H38G, G71N, L83Y, Q112N, V114Q, L116Y, L116I and R124E; Fig. 4a). As a result of the rapid HR responses, these reactions were quantified at 3 d post‐infiltration using fluorescence scanning (Praz *et al*., 2017; Fig. S5), and we observed differences in the effects of each of the mutations on the cell death response by PM3A and PM3F^L456P/Y458H^ (Fig. 4b). The alterations H38G, G71N and L83Y enhanced the cell death response by both PM3A and PM3F^L456P/Y458H^ by two‐ to three‐fold (Fig. 4b). For the Q112N construct, the increase in HR was stronger with PM3A (five‐fold) than with PM3F^L456P/Y458H^ (three‐fold). The opposite was observed for the V114Q, L116Y, L116I and R124E mutations, where the increase in HR was stronger with PM3F^L456P/Y458H^ than PM3A, and up to 12‐fold higher than the wild‐type for the V114Q, L116Y and R124E alterations (Fig. 4b). It is interesting to note that two positions identified as enhancing also gave different results depending on the residue alteration, where the change from histidine to glycine at position 38 enhanced recognition, but the change to glutamine gave no discernible effect (Fig. 4a). Altering leucine at position 116 to tyrosine enhanced recognition by both PM3A and PM3F^L456P/Y458H^ by 12‐fold, whereas changing it to isoleucine specifically enhanced PM3F^L456P/Y458H^ recognition by six‐fold, and showed no effect on recognition by PM3A.

**Figure 5:**
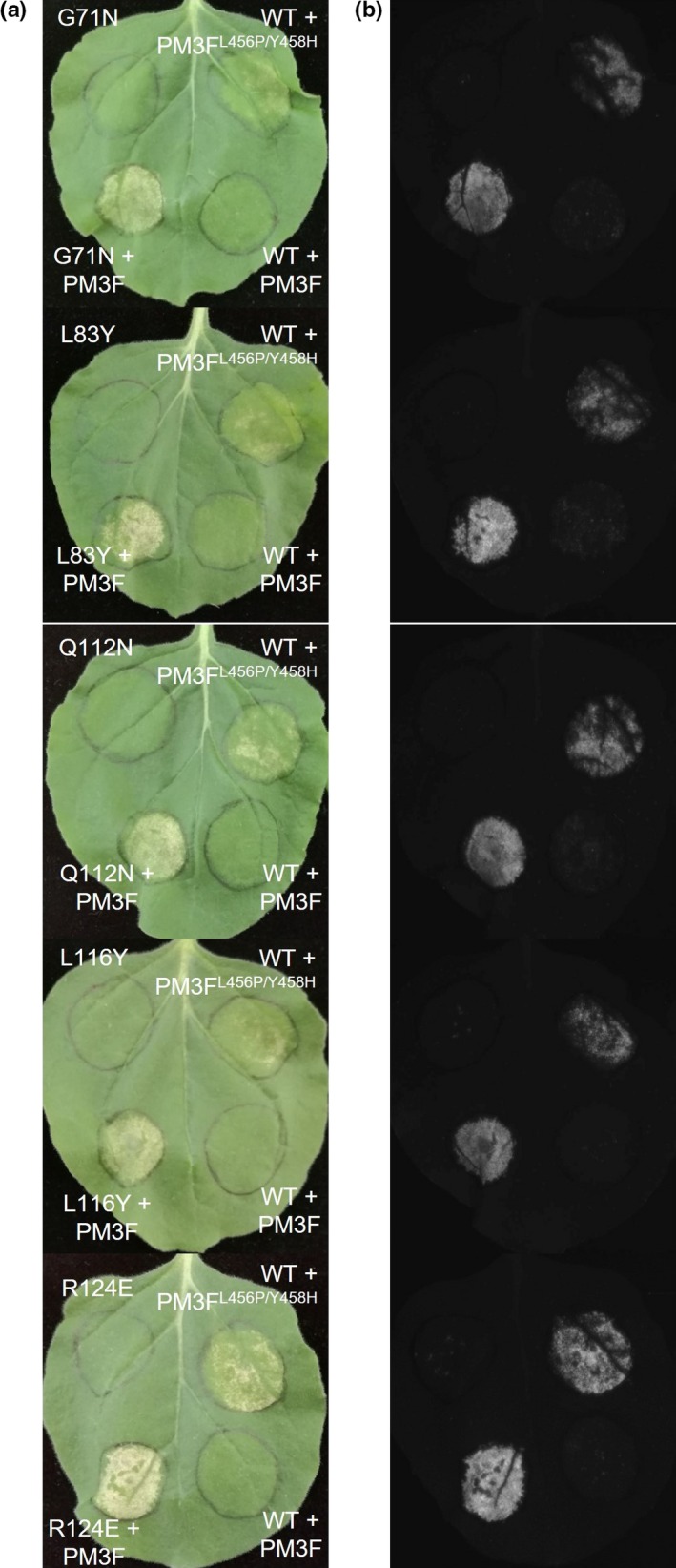
Single‐site mutations in the wheat powdery mildew AVRPM3^A2/F2^ avirulence protein confer a gain of recognition when co‐infiltrated with the natural PM3F allele. (a) Leaf images taken at 3 d post‐infiltration (dpi) demonstrate the strong hypersensitive response (HR) of the five single‐residue alterations that give enhanced recognition with the natural PM3F allele compared with the wild‐type (WT) AVRPM3^A2/F2^. (b) Fluorescence imaging of the leaves visualizes the weak HR of the WT + PM3F control.

Previously, it has been shown that the wild‐type AVRPM3^A2/F2^‐A variant induces only weak and occasional HR when co‐expressed with the natural PM3F in *N. benthamiana* (Bourras *et al*., 2015). Therefore, we tested all single‐residue‐altered constructs with PM3F to observe similarly enhanced recognition with the natural allele. We found five mutations that enhanced recognition with PM3A and PM3F^L456P/Y458H^ and also elicited HR when co‐expressed with PM3F to levels similar to the positive control (G71N, L83Y, Q112N, L116Y and R124E; Fig. 5; Fig. 4a, teal boxes). A complete deletion of the region from Q112N to the L116Y mutation resulted in a disruption of recognition by PM3A, PM3F and PM3F^L456P/Y458H^ (Fig. S6). Surprisingly, two mutations which gave a 12‐fold increase in the response by PM3F^L456P/Y458H^ (L116Y and R124E) also elicited HR with PM3F; however, the Q112N alteration that was also recognized by natural PM3F was not so strongly recognized by the modified PM3F^L456P/Y458H^, suggesting that the effect of these residues was specifically dependent on the L456P and Y458H modifications in the NBS domain of the PM3F^L456P/Y458H^ protein.

We conclude that the cell death response following recognition of the AVRPM3^A2/F2^ effector by PM3A, PM3F and the modified PM3F^L456P/Y458H^ receptor is highly sensitive to single‐residue alterations in AVRPM3^A2/F2^. We observed differences across the protein, including a neutral region from residues 60 to 75, and a concentration of residue alterations resulting in an enhanced cell death response in the region from residue 112 to 116.

### Comparing residues under selection pressure with the effects of single‐residue alterations on recognition by PM3A/F distinguishes putative AVRPM3^A2/F2^ functional domains

To determine whether selection to evade recognition is a dominant driver of evolution among the *AvrPm3* ^*a2/f2*^ haplotypes, we analyzed the selection acting on the *AvrPm3* ^*a2/f2*^ gene and the *AvrPm3* ^*a2/f2*^ effector family. Among the 13 *AvrPm3* ^*a2/f2*^ haplotype sequences are a 6‐bp insertion and 23 single nucleotide polymorphisms (SNPs), all but one of which are nonsynonymous (Table S8). Excluding the *AvrPm3* ^*a2/f2*^ *‐G* haplotype, which is probably the result of a gene conversion event, the 278.4 nonsynonymous and 89.3 synonymous sites represent a significant excess of nonsynonymous substitutions relative to neutral expectation (*P *= 0.016). Tests for positive selection within the gene family using the Bayes empirical Bayes procedure (Yang *et al*., 2005) to compare M8a and M8 models (see the Materials and Methods section) identified 11 positions under significant positive selection (Fig. 4a, green boxes). This region of the protein (residues 32–59) shares no overlap with the domains identified by single‐residue alterations as being distinctly neutral (residues 60–75) or enhancing recognition (residues 112–116). The positively selected residues also do not share substantial overlap with natural polymorphisms in *AvrPm3* ^*a2/f2*^ (Fig. 4a, black framed boxes). The single exception in both cases is position 38, where some haplotypes encode a neutral alteration to glutamic acid (Q). The alteration to glycine (G) at this position, which is common within the effector family but was not found among natural AVRPM3^A2/F2^ variants, caused two‐fold HR enhancement with PM3F^L456P/Y458H^ and three‐fold HR enhancement with PM3A (Fig. 4b).

Thus, we have identified a region under positive selection in the effector family (residues 32–59) and a protein domain where natural polymorphisms are concentrated (residues 86–95). Both regions are distinct from the two functionally identified domains, including the ‘enhancing’ region (residues 112–116), which might constitute an interaction domain. These data indicate that positive selection acting on the *AvrPm3* ^*a2/f2*^ effector family is distinct from the selection pressure driving diversity of *AvrPm3* ^*a2/f2*^ in natural isolates. This suggests that mutations to evade recognition by *Pm3a* and *Pm3f* would not necessarily interfere with effector function.

## Discussion

### Maintenance of a recognized effector at high frequencies in natural populations is possibly caused by the presence of a suppressor

The characterization of the natural diversity of fungal or oomycete avirulence genes has generally been limited to relatively small sample sizes (Allen *et al*., 2008; Barrett *et al*., 2009; Kanzaki *et al*., 2012), or has been primarily marker based (Parlange *et al*., 2009; Oliva *et al*., 2015), a strategy which focuses exclusively on known polymorphisms and therefore cannot detect novel diversity. Our genotyping approach of sequencing complete genes enabled us to comprehensively identify the natural diversity of *AvrPm3* ^*a2/f2*^, globally. From this large collection, we could observe that the recognized AVRPM3^A2/F2^‐A variant is maintained at high frequencies in all regions despite the presence of *Pm3a/f* wheat in cultivation. This contrasts with studies of *AvrLm4‐7* diversity following prolonged exposure of a population of *L. maculans* to selection pressure from *Rlm7* resistance (Daverdin *et al*., 2012). During the course of 4 yr, the frequency of avirulent *AvrLm7* alleles sharply decreased and, accordingly, *Rlm7*‐mediated resistance was all but defeated (Daverdin *et al*., 2012). In our study, we observed the recognized AVRPM3^A2/F2^‐A variant at high frequencies, and many of these isolates also encoded the previously functionally validated variant of the suppressor of recognition, *SvrPm3* ^*a1/f1*^ *‐A* (Bourras *et al*., 2015; Table 4; Fig. S3). The association observed between active expressed suppressor and virulence on *Pm3a* and *Pm3f* in these isolates fits with a model in which the quantitative action of *SvrPm3* ^*a1/f1*^ shelters *AvrPm3* ^*a2/f2*^ from recognition by *Pm3a* and *Pm3f*.

However, our data also show that there are a considerable number of isolates that defy the three‐component *Avr‐R‐Svr* model (Table 4). First, we observed 24 isolates that do not encode the recognized AVRPM3^A2/F2^‐A variant, but that nevertheless exhibit avirulence on *Pm3a*,* Pm3f* or both (Table 2). More than one‐half of these isolates (14) encode the AVRPM3^A2/F2^‐F variant and were originally collected on wild emmer wheat, hinting at the possible association between host diversity at the crop center of origin and pathogen diversity (Ben‐David *et al*., 2016). They have a genetic background closely related, but distinct from, isolates collected on domestic durum or bread wheat. There are probably additional factors in this genetic background that restrict virulence on the domesticated *Pm3a/f* wheat cultivars. The other ten exceptions might be explained by the presence of a second avirulence factor in the pathogen, recognized by *Pm3a*. This *AvrPm3* ^*a3*^ was previously genetically identified in the cross of two European isolates (Bourras *et al*., 2015), where segregating phenotypes in the progeny implied an additional recognized factor. It is likely that this factor is polymorphic in natural isolates, and leads to avirulent phenotypes in the absence of a recognized AVRPM3^A2/F2^ variant.

In addition, we observed 16 isolates encoding AVRPM3^A2/F2^‐A and an SVRPM3^A1/F1^ variant other than the functionally validated SVRPM3^A1/F1^‐A suppressor that exhibited some level of virulence on *Pm3a*,* Pm3f* or both (Table 4). It is as yet unclear whether alternative variants of SVRPM3^A1/F1^, such as the ‘B’ variant, function as active suppressors. Follow‐up studies testing the suppressor activity of these variants and expanding expression studies to natural isolates from populations outside of Europe should determine the variability in suppressor activity of sequence‐diverse variants and in natural populations globally.

### Information from effector family sequence diversity can inform studies of recognition specificity

Previous mutagenesis approaches to investigate the basis of recognition specificity have focused primarily on limited polymorphisms in the natural diversity (Allen *et al*., 2008; Parlange *et al*., 2009; Kanzaki *et al*., 2012) or random mutagenesis (Leonelli *et al*., 2011), or have relied on information from already solved protein structures (Chou *et al*., 2011; Blondeau *et al*., 2015; Maqbool *et al*., 2015). To reduce bias in selecting additional residue alterations outside the natural allelic diversity of the *AvrPm3* ^*a2/f2*^ gene, we identified the most common residue at each position within the structurally related, sequence‐divergent family. Comparatively, alanine screens have been widely used to test the role of specific residues in function and recognition (Joosten & de Wit, 1999; Whisson *et al*., 2007). In theory, alanine screening answers the question of the specific role of an amino acid side‐chain, but the drastic alteration of the biochemical properties of residues is likely to also affect protein structure and stability. By contrast, by selecting common residues within the gene family towards which we altered single residues in the AVRPM3^A2/F2^ protein, we reduced the chances of disturbing the overall structure of the protein. This is an important aspect to consider in a large‐scale mutant screen in which the measurement of protein stability for each tested construct is not feasible. This is particularly crucial for studies of the AVRPM3^A2/F2^ protein, as attempts to tag the protein whilst maintaining functionality have thus far been unsuccessful. Ultimately, testing residues common within the effector family, which represents the pool of functional diversity available to the effector, not only reduces the probability of selecting residue alterations that may disrupt overall protein structure, but is also informative with regard to the specificity of the receptor for the *AvrPm3* ^*a2/f2*^ gene.

Utilizing an effector family‐informed mutagenesis approach to study recognition specificity allowed us to identify single‐residue alterations that enhance the HR on recognition by the cognate R protein. To our knowledge, an enhanced HR resulting from alterations in the AVR protein has not been described previously. None of the enhancing residue alterations we identified exist as polymorphisms in the natural diversity of the *AvrPm3* ^*a2/f2*^ gene. Thus, a study limited to the natural sequence diversity, similar to *AvrLm4‐7* (Blondeau *et al*., 2015), *AvrL567* (Ravensdale *et al*., 2012) and *ATR1* (Krasileva *et al*., 2010; Chou *et al*., 2011), would have found no enhanced recognition effects.

In addition, compared with random mutagenesis, our approach provided clues to which of the shared structural characteristics within the family are important for recognition. It enabled us to identify even conservative residue changes in the AVRPM3^A2/F2^ protein that affect recognition specificity, and also revealed unforeseen variation in the strength of the immune response by *Pm3a* and *Pm3f*. This was exemplified by the single biochemically conservative L116I alteration that resulted in a 10‐fold increase in the HR elicited by the PM3F^L456P/Y458H^ construct. It is possible that disruption and enhancement of the HR may be related to protein stability; however, attempts to tag a functional AVRPM3^A2/F2^‐A have thus far been unsuccessful. Furthering our understanding of the role of specific residues in recognition will be a useful tool for the potential development of synthetic *R* genes with enhanced recognition abilities. Future studies of residues in the NLR that interact with the functional residues we have identified in this study might allow us to design a PM3 variant that recognizes the most common features or residues shared by the *AvrPm3* ^*a2/f2*^ family. The development of NLRs with such expanded structural recognition features would be a robust source of resistance in breeding.

### Analysis of additional sequence diversity can distinguish effector function domains from sites determining recognition specificity

**Figure 6:**
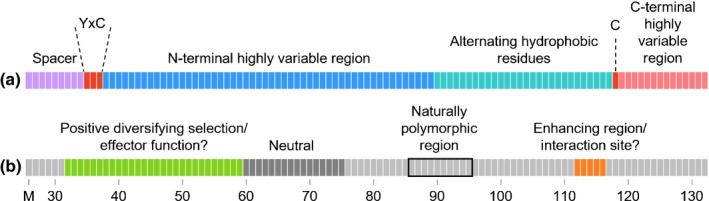
Overview of structural and functional domains of the wheat powdery mildew AVRPM3^A2/F2^ avirulence protein. Schematic depiction of (a) structural domains conserved among the AVRPM3^A2/F2^ effector family adapted from Bourras *et al*. (2016), and (b) the functional domains identified in this study.

By characterizing the natural diversity, testing single‐residue alterations for recognition and estimating selection acting on the effector family, we have identified four distinct domains in the AVRPM3^A2/F2^ protein (Fig. 6b): (1) residues under positive diversifying selection in the effector family (residues 32–59; Fig. 6b, green), which overlap with the highly variable region and the YxC motif conserved in the effector family (Fig. 6a); (2) the neutral domain (residues 60–75; Fig. 6b, dark gray), which also overlaps with the highly variable region; (3) the domain that is most polymorphic in the *AvrPm3* ^*a2/f2*^ natural alleles (Fig. 6b, black frame); and (4) the domain in which residue alterations enhanced and/or altered the specificity of the HR elicited by PM3A/F (residues 112–116; Fig. 6b, orange). Assuming direct recognition of AVRPM3^A2/F2^ by PM3A/F, the enhancing domain might constitute a putative interaction site. Traditionally, selection pressure from direct recognition by the NLR drives *Avr* diversification, often at the site of their protein–protein interactions (Ravensdale *et al*., 2012; Maqbool *et al*., 2015); however, we observed no naturally occurring polymorphisms at the putative interaction site. We did observe that most of the alterations in the naturally polymorphic domain disrupted recognition, but it is unclear why this region shows a high level of diversification or whether this diversity is related to recognition, as most residue alterations across the protein are sufficient to disrupt recognition (Fig. 4a, black boxes). As both of these domains are distinct from the domain under positive selection in the effector family, we conclude that the latter is probably involved in effector function, perhaps co‐evolving with a target protein in the host.

In a model of indirect recognition, mutations that enhance effector function would increase the signal of a modified decoy or guardee that is detected by PM3A/F, leading to a stronger HR. Alternatively, if the effector function of AVRPM3^A2/F2^ involves suppression of host defense responses, and this activity is maintained in the *N. benthamiana* system, mutations in residues important for this virulence function might reduce the ability of the effector to suppress the HR, but still allow for PM3A/F recognition. This would effectively enhance the visible HR in transient assays. Increased HR could also be the result of a change in the rate of effector protein turnover. Fine tuning of the molecular interaction of an R protein that directly recognizes a cognate AVR or enhancing the indirect recognition of modifications to effector targets are both potential strategies for the engineering of enhanced resistance in this interaction.

### Selection pressures other than recognition can also drive *Avr* allele diversification

Comparing the number of disruptive alterations based on natural diversity (10/16, 63%) with those designed using information from the effector family (50/74, 67.8%), we observed that polymorphisms identified in naturally occurring AVRPM3^A2/F2^ variants are equally as likely to disturb recognition as a selection of alterations from our screen. This contrasts strongly with the observation that the additive effects of multiple mutations are required to disrupt the direct recognition of *AvrL567* by *L5* and *L6* alleles (Ravensdale *et al*., 2012). In the evolutionary context, one mutation that abolishes recognition is sufficient to eliminate host resistance as a source of selection pressure. Eight of 12 natural AVRPM3^A2/F2^ variants contain the G86E mutation, which is sufficient to disrupt recognition. Six of these variants have additional mutations that alone are sufficient to abolish recognition by PM3A/F (Table 1). The G86E polymorphism is therefore more likely to be ancestral than a gain‐of‐virulence mutation. In the remaining three variants (AVRPM3^A2/F2^‐C, AVRPM3^A2/F2^‐E and AVRPM3^A2/F2^‐D), we find three single polymorphisms (F95L, E93D and E93K, respectively) representing putative gain‐of‐virulence mutations. The remaining six natural polymorphisms most likely either existed before *Pm3a/f* recognition, are the product of random genetic drift or are the result of selection pressure from other sources, such as effector function. This hypothesis is supported by the absence of significant positive selection acting at positions within the putative interaction domain (residues 112–116). We detected, however, significant positive selection at residues in the effector family, which should result from selection for effector function and not recognition. Novel effector functions have been described in *Cladosporium fulvum*, where both *Avr4* and *CfEcp6* effectors bind chitin through their lysine (LysM) domains, but only *Avr4* has been demonstrated to have the additional function of protecting fungal hyphae from hydrolysis by plant enzymes (van den Burg *et al*., 2006; de Jonge *et al*., 2010). All of the mildew isolates tested here encode the *AvrPm3* ^*a2/f2*^ gene, suggesting that loss of the gene might result in fitness costs. In addition, the region experiencing positive diversifying selection in the effector family, characteristic of shared effector function, is not the domain experiencing diversification in the *AvrPm3* ^*a2/f2*^ alleles. We conclude that neither recognition nor shared effector family function, but rather an important novel function that increases pathogen fitness, is potentially the main driver of diversity within the *AvrPm3* ^*a2/f2*^ gene.

## Author contributions

K.E.M., S.B. and B.K. designed the study. K.E.M., L.L. and L.K. performed the experiments. K.E.M., F.M. and C.R.P. performed the analyses. K.E.M., C.R.P, M.C.M, R.B‐D., K.C., A.D. and E.M. collected the data. K.E.M., S.B. and B.K. wrote and edited the manuscript. R.B‐D., C.C., M.X., F.Z., S.G. and D.Y. contributed the data.

## Supporting information

Please note: Wiley Blackwell are not responsible for the content or functionality of any Supporting Information supplied by the authors. Any queries (other than missing material) should be directed to the *New Phytologist* Central Office.

**Fig. S1** Recognition tests for AVRPM3^A2/F2^ variants.
**Fig. S2** Recognition tests for AVRPM3^A2/F2^‐F.
**Fig. S3** Geographic distribution of AVRPM3^A2/F2^–SVRPM3^A1/F1^ variant combinations.
**Fig. S4** Expression analysis of isolates encoding the active AVRPM3^A2/F2^‐A variant and a SVRPM3^A1/F1^ variant with unknown activity.
**Fig. S5** Single‐site mutations in AVRPM3^A2/F2^ that enhance recognition when co‐infiltrated with PM3A and PM3F^L456P/Y458H^.
**Fig. S6** Recognition test of a five‐amino‐acid deletion of the putative enhancing region (residues 112–116) of AVRPM3^A2/F2^‐A.
**Table S1** Primers used for specific amplification of *AvrPm3* ^*a2/f2*^ (pu7) and *SvrPm3* ^*a1/f1*^ (bcg1)
**Table S7** Student's *t*‐test of significant differences in expression
**Table S8** Nucleotide polymorphisms in the *AvrPm3* ^*a2/f2*^ haplotypes
**Notes S1** Genetic evidence indicating that *AvrPm3* ^*a2/f2*^ is not recognized by *Pm1a*,* Pm2*,* Pm3b‐e*,* Pm3g*,* Pm4a*,* Pm4b*,* Pm5a*,* Pm8* and *Pm17*.

**Table S2** Single‐residue‐altered AVRPM3^A2/F2^ constructs

**Table S3** The worldwide collection of mildew isolates, their AVRPM3^A2/F2^ and SVRPM3^A1/F1^ genotypes, and phenotypes on *Pm3a/f* wheat

**Table S4** Phenotypes of progeny from the 96224 × 94202 population on *Pm1a*,* Pm4a* and *Pm4b* wheat

**Table S5** Phenotypes of progeny from the 96224 × 94202 population on *Pm5a* wheat

**Table S6** Phenotypes of progeny from the 96224 × 94202 population on *Pm17* wheat
