# Supplementary material for: Distinct domains of the AVRPM3A2/F2 avirulence protein from wheat powdery mildew are involved in immune receptor recognition and putative effector function
Source: New Phytol. 2018 Feb 17;218(2):681–95. doi: 10.1111/nph.15026 (PMC6175116; doi:10.1111/nph.15026)
Supplement: Supplementary file 1 — Fig. S1 Recognition tests for AVRPM3A2/F2 variants. Fig. S2 Recognition tests for AVRPM3A2/F2‐F. Fig. S3 Geographic distribution of AVRPM3A2/F2–SVRPM3A1/F1 variant combinations. Fig. S4 Expression analysis of isolates encoding the active AVRPM3A2/F2‐A variant and a SVRPM3A1/F1 variant with unknown activity. Fig. S5 Single‐site mutations in AVRPM3A2/F2 that enhance recognition when co‐infiltrated with PM3A and PM3FL456P/Y458H. Fig. S6 Recognition test of a five‐amino‐acid deletion of the putative enhancing region (residues 112–116) of AVRPM3A2/F2‐A. Table S1 Primers used for specific amplification of AvrPm3 a2/f2 (pu7) and SvrPm3 a1/f1 (bcg1) Table S7 Student's t‐test of significant differences in expression Table S8 Nucleotide polymorphisms in the AvrPm3 a2/f2 haplotypes Notes S1 Genetic evidence indicating that AvrPm3 a2/f2 is not recognized by Pm1a, Pm2, Pm3b‐e, Pm3g, Pm4a, Pm4b, Pm5a, Pm8 and Pm17. [file NPH-218-681-s001.pdf]

New Phytologist **Supporting Information**

Article title: **Distinct domains of the AVRPM3$^{A2/F2}$ avirulence protein from wheat powdery mildew are involved in immune receptor recognition and putative effector function**

Authors: Kaitlin Elyse McNally, Fabrizio Menardo, Linda Lüthi, Coraline Rosalie Praz, Marion Claudia Müller, Lukas Kunz, Roi Ben-David, Kottakota Chandrasekhar, Amos Dinoor, Christina Cowger, Emily Meyers, Mingfeng Xue, Fangsong Zeng, Shuanjun Gong, Dazhao Yu, Salim Bourras and Beat Keller

Article acceptance date: 05 January 2018

The following Supporting Information is available for this article:

Figure S1. Recognition tests for AVRPM3$^{A2/F2}$ variants.

Figure S2. Recognition tests for AVRPM3$^{A2/F2}$-F.

Figure S3. Geographic distribution of AVRPM3$^{A2/F2}$-SVRPM3$^{A1/F1}$ variant combinations.

Figure S4. Expression analysis of isolates encoding the active AVRPM3$^{A2/F2}$-A variant and a SVRPM3$^{A1/F1}$ variant with unknown activity.

Figure S5. Single site mutations in AVRPM3$^{A2/F2}$ that enhance recognition when co-infiltrated with PM3A and PM3F$^{L456P/Y458H}$.

Figure S6. Recognition test of a 5 amino acid deletion of the putative enhancing region (residues 112-116) of AVRPM3$^{A2/F2}$-A.

Table S1. Primers used for specific amplification of *AvrPm3$^{a2/f2}$* (pu7) and *SvrPm3$^{a1/f1}$* (bcg1).

Table S2. Single residue altered AVRPM3$^{A2/F2}$ constructs. (see separate file)

Table S3. The worldwide collection of mildew isolates, their AVRPM3$^{A2/F2}$ and SVRPM3$^{A1/F1}$ genotypes, and phenotypes on *Pm3a/f*. (see separate file)

Table S4. Phenotypes of progeny from the 96224x94202 population on *Pm1a, Pm4a,* and *Pm4b* wheat. (see separate file)

Table S5. Phenotypes of progeny from the 96224x94202 population on *Pm5a* wheat. (see separate file)

Table S6. Phenotypes of progeny from the 96224x94202 population on *Pm17* wheat. (see separate file)

Table S7. Student's t-test of significant differences in expression.

Table S8. Nucleotide polymorphisms in the *AvrPm3*$^{a2/f2}$ haplotypes.

Notes S1. Genetic evidence indicating *AvrPm3*$^{a2/f2}$ is not recognized by *Pm1a, Pm2, Pm3b-e, Pm3g, Pm4a, Pm4b, Pm5a, Pm8,* and *Pm17*.

Figure S1. Recognition tests for AVRPM3$^{A2/F2}$ variants. Transient agrobacterium infiltration assays in *Nicotiana benthamiana* with a 1:4 ratio of R:AVR protein show no interaction between AVRPM3$^{A2/F2}$ variants other than 'A' (positive control) and the PM3A and PM3F$^{L456P/Y458H}$ resistance proteins from wheat. Fluorescence imaging of the leaves taken at 5 dpi reveals HR only with the AVRPM3$^{A2/F2}$ variant 'A' + PM3A or PM3F$^{L456P/Y458H}$ controls.

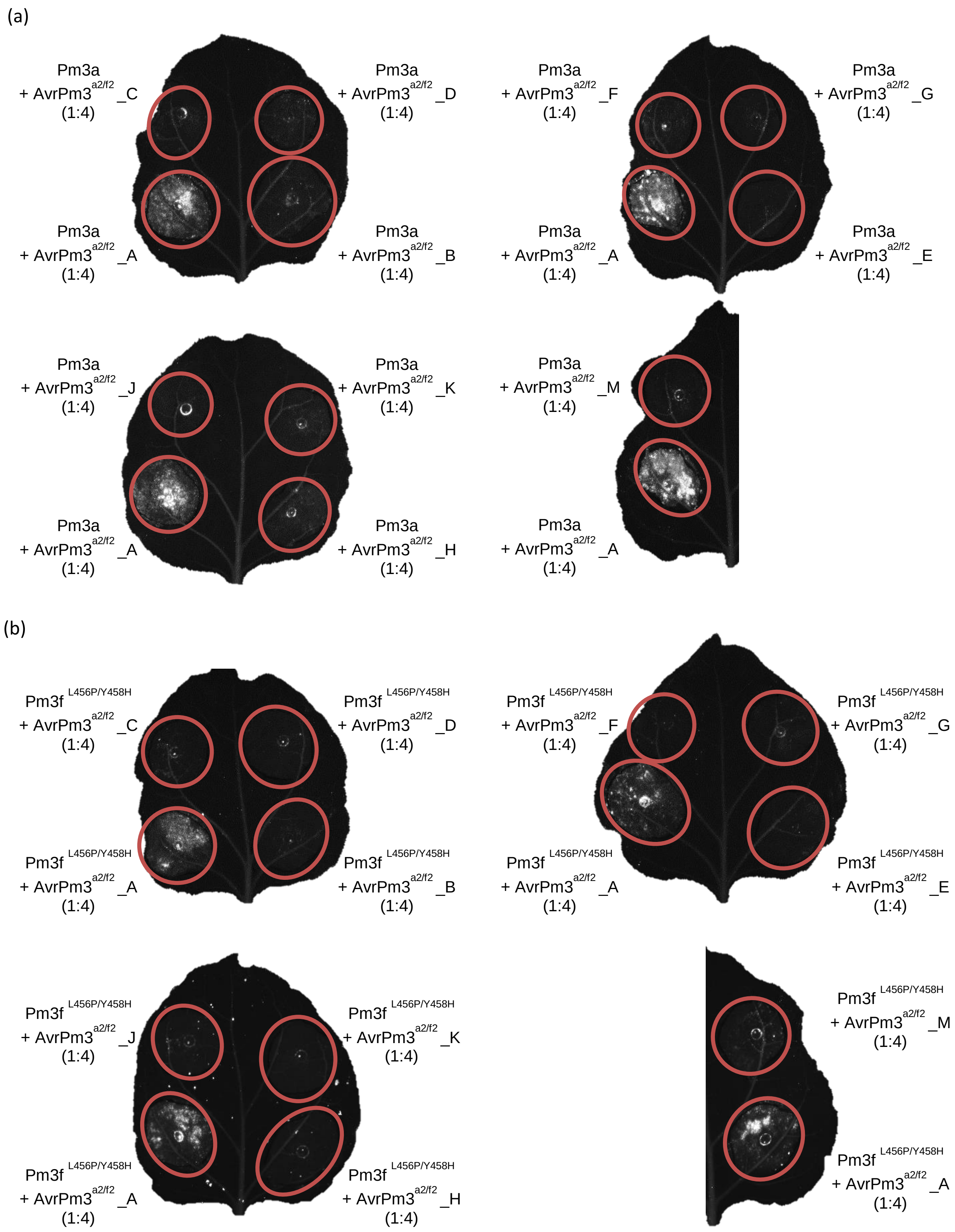

Figure S2. Recognition tests for AVRPM3$^{A2/F2}$-F. Transient agrobacterium infiltration assays in *Nicotiana benthamiana* show no interaction between the AVRPM3$^{A2/F2}$ variant ‘F’ and the PM3A, B, C, D, E, F$^{L456P/Y458H}$, and PM8 resistance proteins from wheat. Fluorescence imaging of the leaves taken at 5 dpi reveals HR only with the AVRPM3$^{A2/F2}$ variant ‘A’ + PM3A control.

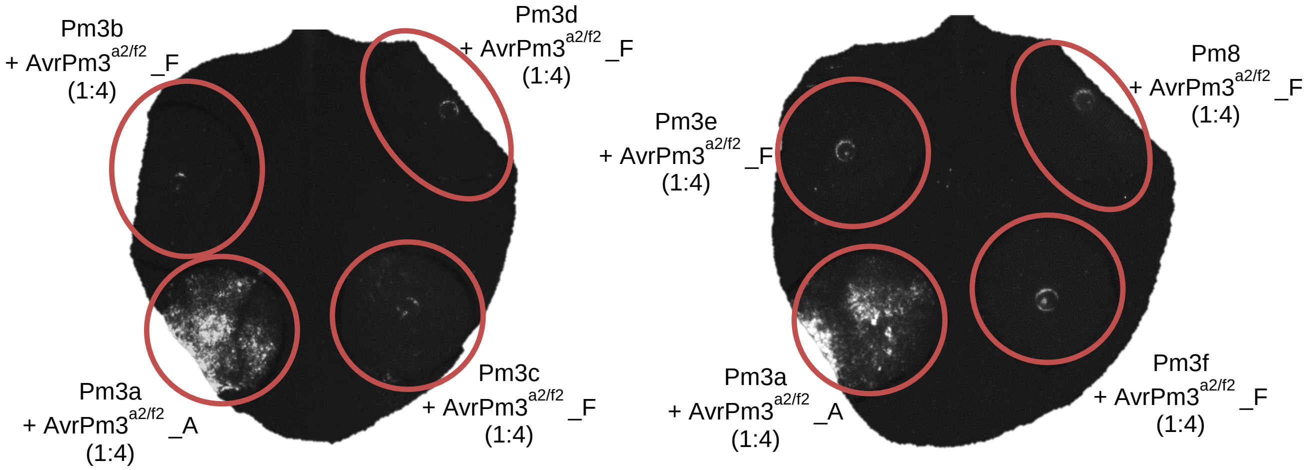

Figure S3. Geographic distribution of AVRPM3$^{A2/F2}$-SVRPM3$^{A1/F1}$ variant combinations. Variant combinations (AVR-SVR) from the US, Europe, Israel, China, Japan, and Australia. Area of the circles correspond to the number of isolates from that region.

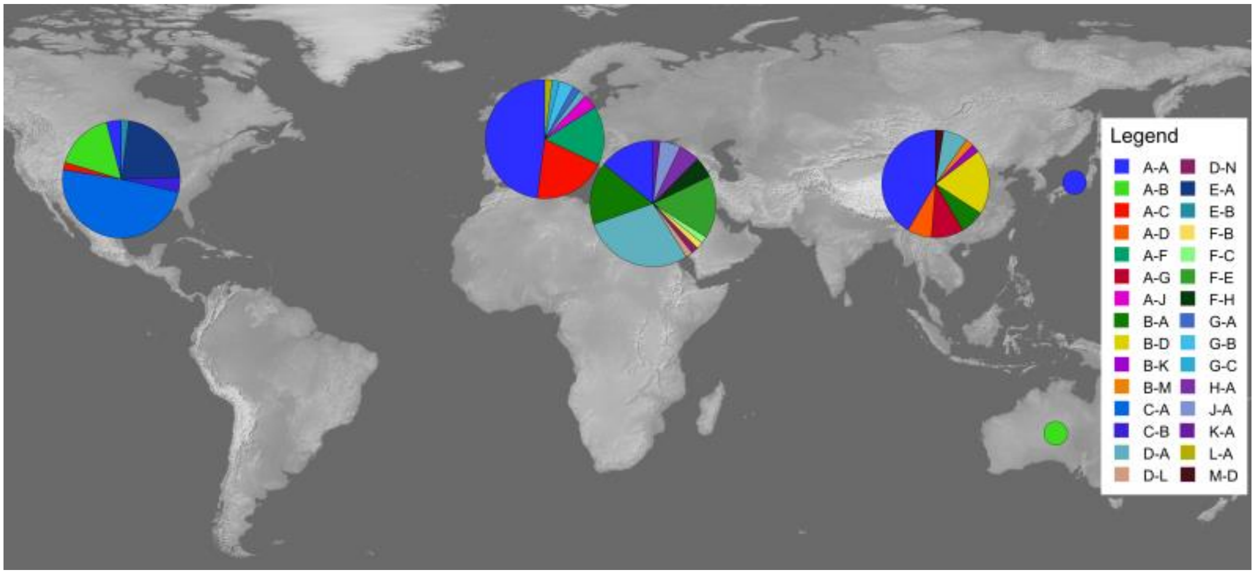

Figure S4. Expression analysis of isolates encoding the active AVRPM3$^{A2/F2}$-A variant and a SVRPM3$^{A1/F1}$ variant with unknown activity. The mean normalized expression of *SvrPm3$^{a1/f1}$* (dark grey histograms) and *AvrPm3$^{a2/f2}$* (light grey histograms) at 2d after infection. Leaf segments from the susceptible recurrent parent line '*Chancellor*' were infected with *B.g. tritici (t)* and *B.g. triticale (T)* isolates from diverse geographic origins (Europe, EU; China, CN; Australia, AU) that contain the active AVRPM3$^{A2/F2}$ and either the SVRPM3$^{A1/F1}$ -C, -F, -G, -J, -B, or -D variant.

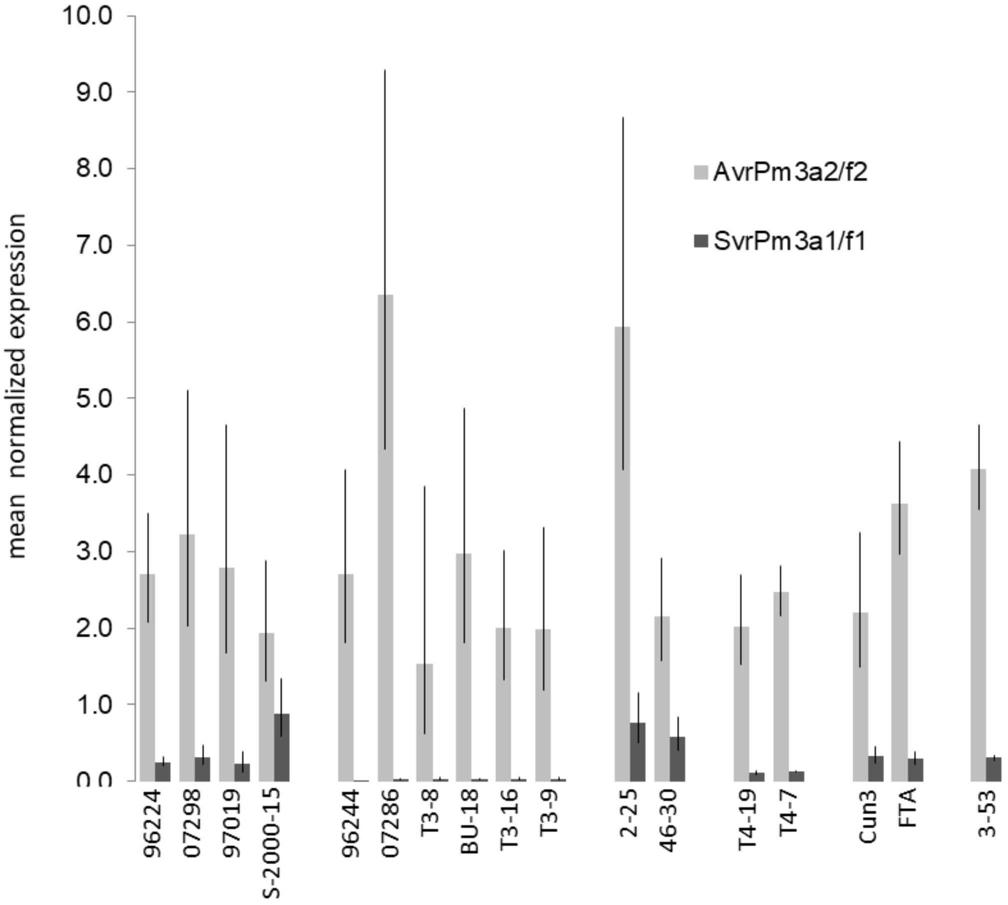

Variant	AVRPM3$^{A2/F2}$	A	A	A	A	A	A	A	A	A	A	A	A	A	A	A	A	A
	SVRPM3$^{A1/F1}$	C	C	C	C	F	F	F	F	F	F	G	G	J	J	B	B	D
Phenotype	*Pm3a*	A	A	A	V	A	A	A	A	A	A	A	I	A	A	A	A	A
	Pm3f	A	A	A	V	A	A	A	I	I	V	V	V	A	A	V	V	V
Formae specialis		*T*	*t*	*t*	*t*	*t*	*t*	*T*	*T*	*T*	*T*	*t*	*t*	*T*	*T*	*t*	*t*	*t*
Origin		EU				EU						CN		EU		AU		CN

Figure S5. Single site mutations in AVRPM3$^{A2/F2}$ that enhance recognition when co-infiltrated with PM3A and PM3F$^{L456P/Y458H}$. Example images of HR quantification assays taken at 2 dpi. *Agrobacteria* expressing the V114Q and L116I altered AVRPM3$^{A2/F2}$ constructs were co-infiltrated in *Nicotiana benthamiana* with PM3A and PM3F$^{L456P/Y458H}$ and these were compared against wildtype AVRPM3$^{A2/F2}$ (WT) with PM3A and PM3F$^{L456P/Y458H}$, respectively.

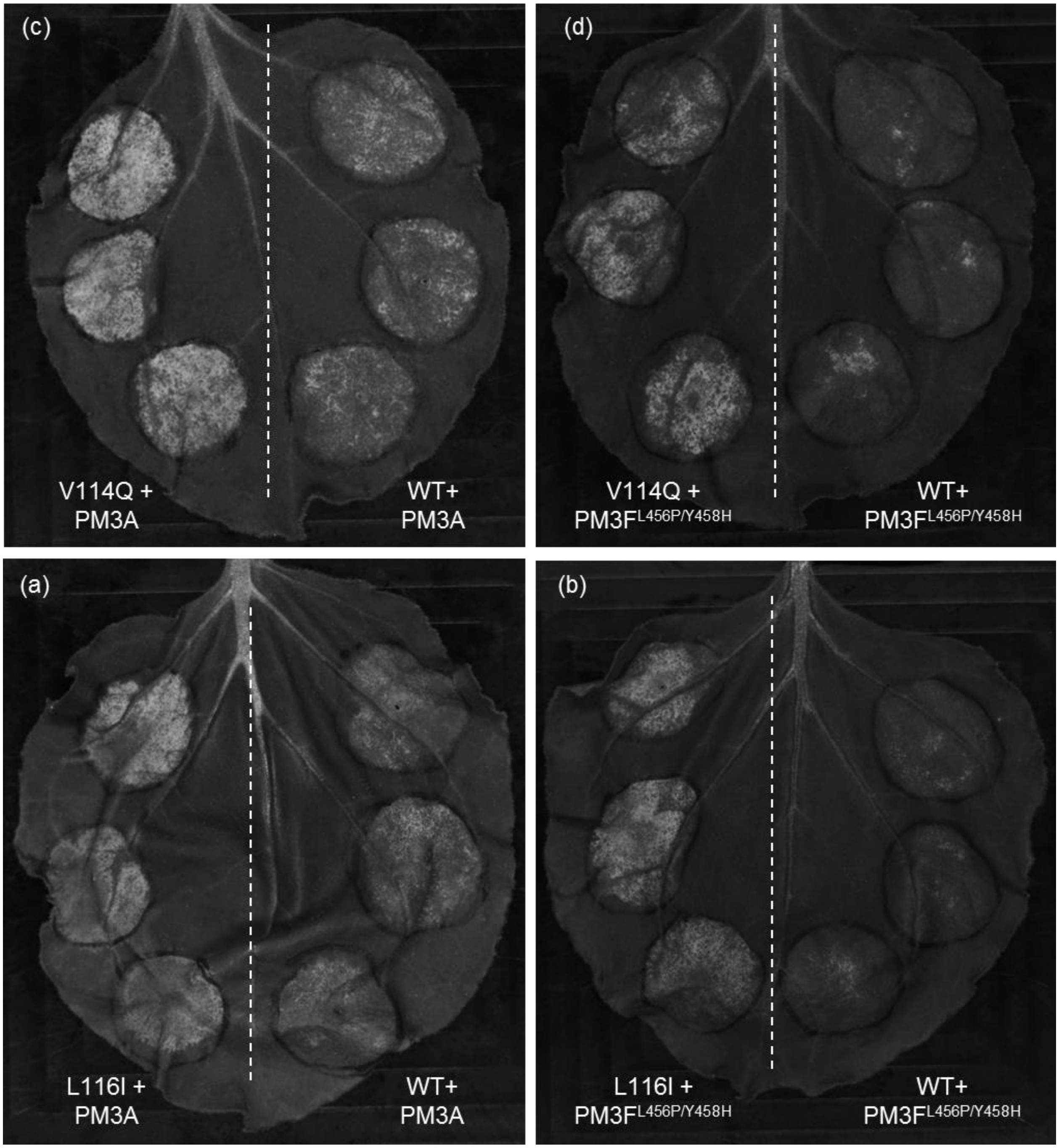

Figure S6. Recognition test of a 5 amino acid deletion of the putative enhancing region (residues 112-116) of AVRPM3$^{A2/F2}$-A. Transient agrobacterium infiltration assays in *Nicotiana benthamiana* show no interaction between the 5 AA deletion mutant (residues 112-116) of AVRPM3$^{A2/F2}$–A and the PM3A or PM3F$^{L456P/Y458H}$ resistance proteins from wheat. Fluorescence imaging of the leaves taken at 5 dpi reveals HR only with the natural AVRPM3$^{A2/F2}$ variant 'A' and the PM3A control.

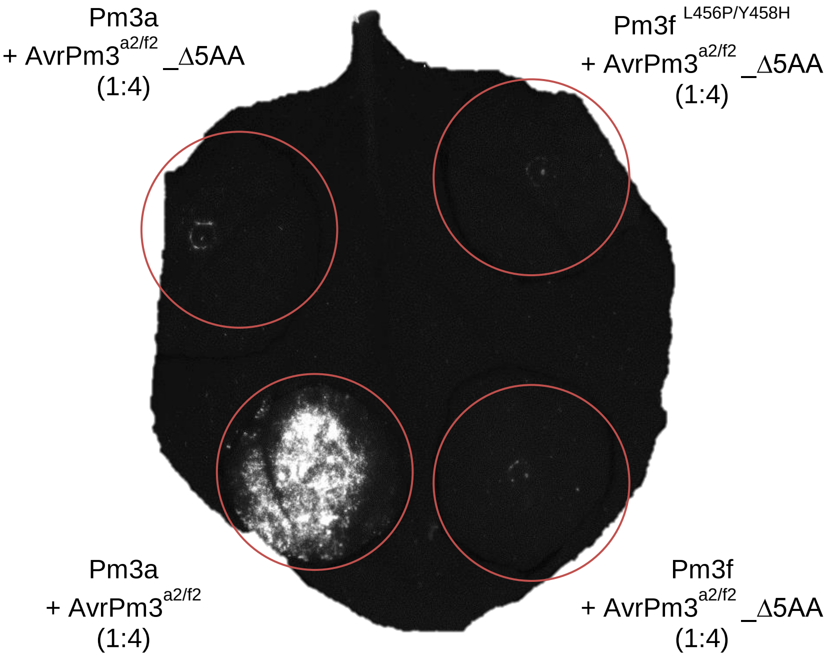

Table S1. Primers used for specific amplification of *AvrPm3*$^{a2/f2}$ (pu7) and *SvrPm3*$^{a1/f1}$ (bcg1).

Primer ID	Sequence
pu7_F2	CCTCTGAACCGCCCCATTT
pu7_R2	CAAATCAGGTCACCCCACCA
bcg1_ext_F	ATGGGTTAGCCTTGACATTCC
bcg1_ext_R	AAGACCAGTCATCCCTTCGAC
bcg1_int_F	CATTTGACTTGATTGATGATGATG
bcg1_int_R	CGTTATCCTTGCTTTACCTAGTGA
Y35G_F	AGTGGCAAGTGTCATGACAGGGTCATTGGT
Y35G_R	CTTGCCACTTGAAGCGTTAGCGACAGG
C37G_F	AAGGGTCATGACAGGGTCATTGGTC
C37G_R	ATGACCCTTGTAACTTGAAGCGTTAGCG
H38Q_F	GGACAGGGTCATTGGTCCGGTTAC
H38Q_R	TGACACTTGTAACTTGAAGCGTTAGC
N80S_F	GTGTAATCTTAAAAAGAGGAGAAGAAAATA
N80S_R	TAAAGTATCTTGAACCAAATTTCTGTCCT
N89Y_F	TATATTAAAGTCGAATTTTTTGTGGGAAT
N89Y_R	TTCTTCTCCTCTTTTTAAGATTACATTAA
K91T_F	CAGTCGAATTTTTTGTGGGAAT
K91T_R	TAATATTTTCTTCTCCTCTTTTTAAGATTACATTA
E93K_F	AAATTTTTTGTGGGAATCAACTATT
E93K_R	GACTTTAATATTTTCTTCTCCTCTTTTTAAGATT
K102H_F	TGCACGAAATTATTTATCTACAGGCTTATGTCC
K102H_R	TCGTGCAAATAGTTGATTCCCACAAAAAATTC
K102A_F	TGGCTGAAATTATTTATCTACAGGCTTATGTCC
K102A_R	TCAGCCAAATAGTTGATTCCCACAAAAAATTC

Primer sequences are written in the 5’ to 3’ orientation.

Table S2. Single residue altered AVRPM3$^{A2/F2}$ constructs.

[Separate excel file]

Table S3. The worldwide collection of mildew isolates, their AVRPM3$^{A2/F2}$ and SVRPM3$^{A1/F1}$ genotypes, and phenotypes on *Pm3a/f* wheat.

[Separate excel file]

Table S4. Phenotypes of progeny from the 96224x94202 population on *Pm1a, Pm4a,* and *Pm4b* wheat.

[Separate excel file]

Table S5. Phenotypes of progeny from the 96224x94202 population on *Pm5a* wheat.

[Separate excel file]

Table S6. Phenotypes of progeny from the 96224x94202 population on *Pm17* wheat.

[Separate excel file]

Table S7. Student's t-test of significant differences in expression.

		7237 *Avr*	7237 *Svr*	JIW2 *Avr*	JIW2 *Svr*	7004 *Avr*	7004 *Svr*	7296 *Avr*	7296 *Svr*
7237	*Avr*	--	0***	0.155	0.001***	0.01*	0.028*	0.003**	0***
7237	*Svr*	--	--	0.006**	0.025*	0***	0.005**	0.003**	0***
JIW2	*Avr*	--	--	--	0.005**	0.082	0.048*	0.015*	0.019*
JIW2	*Svr*	--	--	--	--	0.002*	0.004**	0.005**	0.007**
7004	*Avr*	--	--	--	--	--	0.021*	0.007**	0***
7004	*Svr*	--	--	--	--	--	--	0.007**	0.001**
7296	*Avr*	--	--	--	--	--	--	--	0.419
7296	*Svr*	--	--	--	--	--	--	--	--

*, **, and *** indicate p-values <0.05, 0.01, and 0.001, respectively

Table S8. Nucleotide polymorphisms in the *AvrPm3*$^{a2/f2}$ haplotypes.

AvrPm3a2/f2 Haplotype	62	71	72	73	74	75	76	77	80	87	91	93	114	154	156	175	198	207	239	257	265	272	277	279	285	326	355	365	369	%id
***A*[a]**	**C**	**G**	**-**	**-**	**-**	**-**	**-**	**-**	**G**	**C**	**A**	**C**	**T**	**G**	**A**	**C**	**C**	**G**	**A**	**G**	**A**	**A**	**G**	**A**	**T**	**C**	**T**	**C**	**G**	
A-T	.	.	-	-	-	-	-	-	.	.	.	.	.	.	.	T	.	.	.	.	.	.	.	.	.	.	.	.	.	0.9975
C	.	.	-	-	-	-	-	-	.	.	.	.	.	.	.	.	.	.	.	.	.	.	.	.	G	.	.	.	.	0.9975
D	.	.	-	-	-	-	-	-	.	.	.	.	.	.	.	.	A	.	G	.	.	C	A	.	.	.	.	.	.	0.9898
F	.	.	-	-	-	-	-	-	.	.	.	.	.	.	.	.	A	T	.	A	.	.	.	.	.	.	.	.	.	0.9924
G	T	A	C	T	C	G	C	C	T	T	G	A	G	.	.	.	A	.	.	A	T	.	.	.	.	T	C	.	.	0.9549
M	.	.	-	-	-	-	-	-	.	.	G	A	G	A	T	.	.	.	.	A	.	.	.	.	.	.	.	.	C	0.9822
B	.	.	-	-	-	-	-	-	.	.	.	.	G	.	.	.	.	.	.	A	.	.	.	.	.	.	.	.	C	0.9924
K	.	.	-	-	-	-	-	-	.	.	.	.	G	.	.	.	.	.	.	A	.	.	.	.	.	.	.	.	.	0.9949
H	.	.	-	-	-	-	-	-	.	.	.	.	.	.	.	.	.	.	.	A	.	.	.	.	.	.	.	.	C	0.9949
J	.	.	-	-	-	-	-	-	.	.	.	.	.	.	.	.	.	.	.	A	.	.	.	.	.	.	.	G	C	0.9924
L[a]	.	.	-	-	-	-	-	-	.	.	.	.	.	.	.	.	.	.	.	A	.	.	.	.	.	T	.	.	.	0.9949
E	.	.	-	-	-	-	-	-	.	.	.	.	.	.	.	.	.	.	.	.	.	.	.	T	.	.	.	.	.	0.9975

Nucleotide polymorphisms compared to the avirulent haplotype are depicted. Dashes indicate gaps and dots indicate identical residues. The 'A-T' haplotype was found only in *B.g. triticale* isolates and contains the only synonymous mutation in the haplotypes.

[a]Bourras et al, 2015

Notes S1. Genetic evidence indicating *AvrPm3$^{a2/f2}$* is not recognized by *Pm1a, Pm2, Pm3b-e, Pm3g, Pm4a, Pm4b, Pm5a, Pm8,* and *Pm17.*

In order to assess if *AvrPm3$^{a2/f2}$* is genetically controlling avirulence to other *R* genes, we compared phenotype segregation data on *Pm3f* to those on *Pm1a*, *Pm4a*, *Pm4b*, and *Pm5a* in a subset of haploid progeny originating from a cross between the mildew isolates 96224 and JIW2 (Parlange et al., 2015, Supporting Information Table S4 and S5). If avirulence on *Pm1a*, *Pm4a*, *Pm4b*, or *Pm5a* is controlled by the same genetic locus as *Pm3f*, we expect that the progeny will have the same segregation pattern on *Pm3f* as compared to *Pm1a*, *Pm4a*, *Pm4b*, or *Pm5a*. For *Pm1a*, *Pm4a*, and *Pm4b*, 32 progeny were tested and the phenotype segregation pattern were completely different to those on *Pm3f* (Supporting Information Table S4). For the phenotypes on *Pm5a*, 50 progeny were tested, and phenotype segregation was also completely different to that on *Pm3f* (Supporting Information Table S5). This data indicates that avirulence on *Pm3f* is genetically unlinked to avirulence on *Pm1a*, *Pm4a*, *Pm4b, and Pm5a*. Therefore, it is unlikely that *AvrPm3$^{a2/f2}$* is also recognized by *Pm1a*, *Pm4a*, *Pm4b,* or *Pm5a*.

For *Pm17,* we used the same approach in a subset of 56 haploid progeny originating from a cross between the mildew isolates 96224 and 94202 (Bourras et al. 2015; Supporting Information Table S6). Here also, phenotype segregation on *Pm17* and *Pm3f* were completely different, indicating that avirulence on *Pm3f* is genetically unlinked to avirulence on *Pm17*. Therefore, it is unlikely that *AvrPm3$^{a2/f2}$* is also recognized by *Pm17*.

To conclude, phenotype segregation data from Parlange et al. (2015), Bourras et al. (2015), and Supporting Information Tables S4-S6, as well as genetic mapping data from Parlange et al. (2015), Bourras et al. (2015), and Praz et al. (2017), indicate that *AvrPm3$^{a2/f2}$* is not controlling avirulence towards *Pm1a*, *Pm2*, *Pm3b-e*, *Pm3g*, *Pm4a*, *Pm4b*, *Pm5a*, *Pm8*, and *Pm17*.

References

Bourras S, McNally KE, Ben-David R, Parlange F, Roffler S, Praz CR, Oberhaensli S, Menardo F, Stirnweis D, Frenkel Z, Schaefer LK, Flükiger S, Treier G, Herren G, Korol AB, Wicker T, Keller B. 2015. Multiple avirulence loci and allele-specific effector recognition control the Pm3 race-specific resistance of wheat to powdery mildew. *The Plant Cell* **27:** 2991-3012.

Parlange F, Roffler S, Menardo F, Ben-David R, Bourras S, McNally KE, Oberhaensli S, Stirnweis D, Buchmann G, Wicker T, Keller B. 2015. Genetic and molecular characterization of a locus involved in avirulence of *Blumeria graminis* f. sp. *tritici* on wheat *Pm3* resistance alleles. *Fungal Genetics and Biology* **82:** 181-192.

Praz CR, Bourras S, Zeng F, Sánchez-Martín J, Menardo F, Xue M, Yang L, Roffler S, Böni R, Herren G, McNally KE, Ben-David R, Parlange F, Oberhaensli S, Flükiger S, Schäfer LK, Wicker T, Keller B. 2017. *AvrPm2* encodes an RNase-like avirulence effector which is conserved in the two different specialized forms of wheat and rye powdery mildew fungus. *New Phytologist* **213:** 1301-1314.
